# A systematic review of routing attacks detection in wireless sensor networks

**DOI:** 10.7717/peerj-cs.1135

**Published:** 2022-10-21

**Authors:** Zainab Alansari, Nor Badrul Anuar, Amirrudin Kamsin, Mohammad Riyaz Belgaum

**Affiliations:** 1 Faculty of Computer Science and Information Technology, University of Malaya, Kuala Lumpur, Malaysia; 2 College of Computing and Information Sciences, University of Technology and Applied Sciences, Muscat, Sultanate of Oman; 3 Malaysian Institute of Information Technology (MIIT), Universiti Kuala Lumpur, Kuala Lumpur, Malaysia; 4 Computer Science and Engineering, G. Pullaiah College of Engineering and Technology, Kurnool, India

**Keywords:** Wireless sensor networks, Routing attacks detection, Internet of things, Wormhole attack, Blackhole attack, Grayhole attack, Sinkhole attack, Sybil attack, Hello flood attack, Spoofing attack

## Abstract

Wireless sensor networks (WSNs) consist of hundreds, or thousands of sensor nodes distributed over a wide area and used as the Internet of Things (IoT) devices to benefit many home users and autonomous systems industries. With many users adopting WSN-based IoT technology, ensuring that the sensor’s information is protected from attacks is essential. Many attacks interrupt WSNs, such as Quality of Service (QoS) attacks, malicious nodes, and routing attacks. To combat these attacks, especially on the routing attacks, we need to detect the attacker nodes and prevent them from any access to WSN. Although some survey studies on routing attacks have been published, a lack of systematic studies on detecting WSN routing attacks can be seen in the literature. This study enhances the topic with a taxonomy of current and emerging detection techniques for routing attacks in wireless sensor networks to improve QoS. This article uses a PRISMA flow diagram for a systematic review of 87 articles from 2016 to 2022 based on eight routing attacks: wormhole, sybil, Grayhole/selective forwarding, blackhole, sinkhole, replay, spoofing, and hello flood attacks. The review also includes an evaluation of the metrics and criteria used to evaluate performance. Researchers can use this article to fill in any information gaps within the WSN routing attack detection domain.

## Introduction

Wireless sensor networks (WSNs) use various emerging IoT technologies, have limited infrastructure, and must maintain security while being connected to an unreliable internet (Alansari et al., 2018). WSNs are susceptible to a variety of routing attacks, which are classified according to their characteristics and behaviors. Internal *vs* external attacks compensate the first category. An outsider node disrupts the network during an external attack, whereas an insider node with a valid identity does the same during an internal attack (Fang et al., 2020). The second category is physical attack *vs* remote attack. In a physical attack, the sensor node is physically present, and its hardware could sustain various damages. In a remote attack, however, the attacker must transmit a powerful signal from considerable distances to reach the node. The third category is the active attack *vs* the passive attack. In passive attacks, the attacker node listens and monitors the data at the network level. In contrast, in an active attack, the attacker node targets the network in several ways, such as by generating or removing data.

Using a method to identify abnormal behaviors is one of the best ways to establish security and reliability in WSNs (Alansari et al., 2017). In this respect, anomaly-based intrusion detection systems are considered as one of the main approaches to achieve this goal. A comprehensive survey was presented by Bhushan & Sahoo (2018) on security issues as well as protection techniques designed to defend against malicious attacks in WSNs. They discussed methods of identification and detection alongside countermeasures of many powerful attacks on WSNs, such as sybil attack, DoS attack, wormhole attack and sinkhole attack. Their article examines potential security threats in different protocol layers and does not focus on network layer and routing attacks, some recent detection mechanisms such as rank-based, rule-based, beacon-based, and fuzzy logic methods are lacking. Mohsin (2017) presented a study of routing attacks on the design of WSNs to find out the aim of attackers. The article classifies and compares the routing attacks systematically based on the various characteristics including objectives, the nature of attacks, attack mechanism, WSN target site and route interruption or resource utilization. However, the article only discusses about attacks and lacks detection methods. Similarly, Ioannou & Vassiliou (2016) introduced packet drop attacks on a routing layer and studied the effect of the attacks as “seen” from the sink node and target node. They show that all network layers of the target node are infected by attacks and the degree of effect depends on several factors, including WSN topology. Thus, the article did not discuss about detection methods of routing attacks which is its limitation.

This systematic literature review aims to conduct a comprehensive analysis of the status of routing attack detections in WSNs and provide a new WSN’s taxonomy, characteristics, and functionality along with some discussions on diverse types of attacks.

In this context, the primary objective of this article is to preserve understanding of different WSN routing attacks with their detection method. Furthermore, the current classification of various approaches to detect routing attack is presented in line with the review of literature.

This article significantly expands the dimensions of discussions, widening the scope of the literature review. Therefore, our significant contribution on this review article is:

- Present a systematic review of the literature on routing attack detection techniques in WSNs.
- Discuss the taxonomy of current trends in WSN detection techniques, emphasizing their advantages and disadvantages.
- Characterize the metrics used to measure the efficacy of recent methods.
- Propose future research topics and provide some recommendations for current and future research.

In some applications, WSN security issues cause financial and privacy problems. Consequently, the security of WSNs has recently become a topic of high-level research. WSN’s weak nodes located in an environment can be targeted and attacked easily. The ability to measure and store nodes efficiently tends to result in packet loss or low productivity due to energy constraints. To overcome the above issues, a robust routing attack detection must be designed that considers different performance metrics and uses the best method. Compared with current research, to the best of our knowledge, this study is the first to address advanced SLR frameworks in routing attack detection. Current similar review articles do not cover all twenty-four performance evaluation metrics or different methods that are used to develop routing attack detections for WSN. Moreover, current studies do not cover the relationship between diverse types of attacks, performance evaluation metrics and methods. Therefore, there is an urgent need for a comprehensive SLR on different routing attack detection systems. The intended audiences of this SLR are wireless sensor network administrators, service providers, end-users, and researchers who are willing to propose a method of attack detection or undertake additional research in the future to improve WSN security.

The objective of this SLR is to serve as a foundation for future research. The evaluation’s aim is to analyze and comprehend routing attack detection techniques in WSNs. This is essential if more viable methods to improve current techniques or benefit from previous studies are to be developed. The next tentative brief section of the review is a formal statement that expands through the sections. Section 2 discusses the background of network layer attacks and suggest a possible solution for each attack. Section 3 establishes the methodology used in this article, while Section 4 describes the results, evaluates the hypotheses, discusses the various articles published by classifying the current detection based on different criteria, and finally brings forward the research trends and open issues in the field of WSN, while Section 5 summarizes the SLR and provides recommendations for further research.

## Background

**Figure 1:**
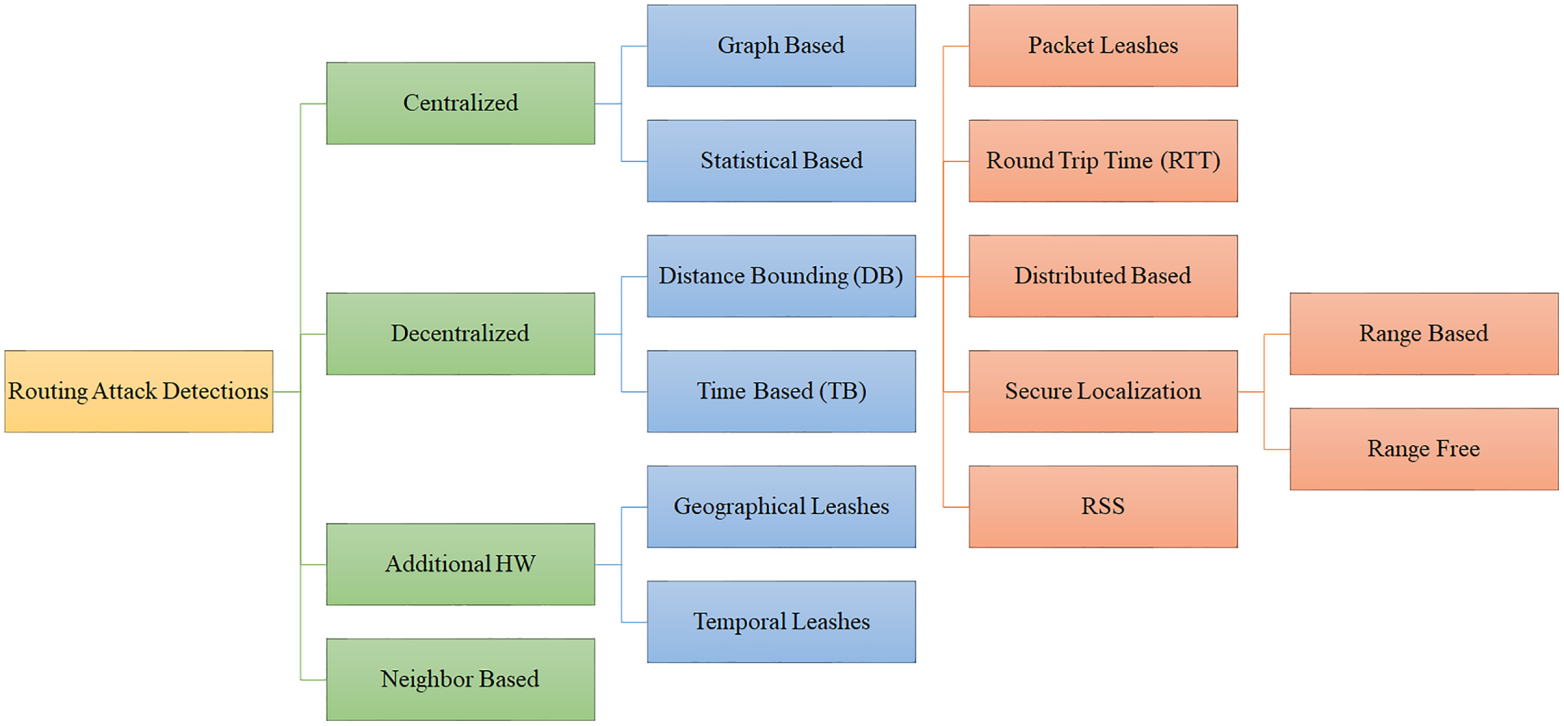
Different type of attacks in WSNs.

The routing protocols are frequently vulnerable to attack because they are typically straightforward. Eight of the most significant routing attacks are wormhole, Sybil, Grayhole/Selective Forwarding, Blackhole, Sinkhole, Replay, Spoofing, and Hello Flood attacks. Below is a detailed explanation of each attack, including its strength and motivation. Additionally, each attack’s severity and implications are discussed. Figure 1 shows various routing attack detections in WSN that were examined in this study.

### Wormhole attacks

**Figure 2:**
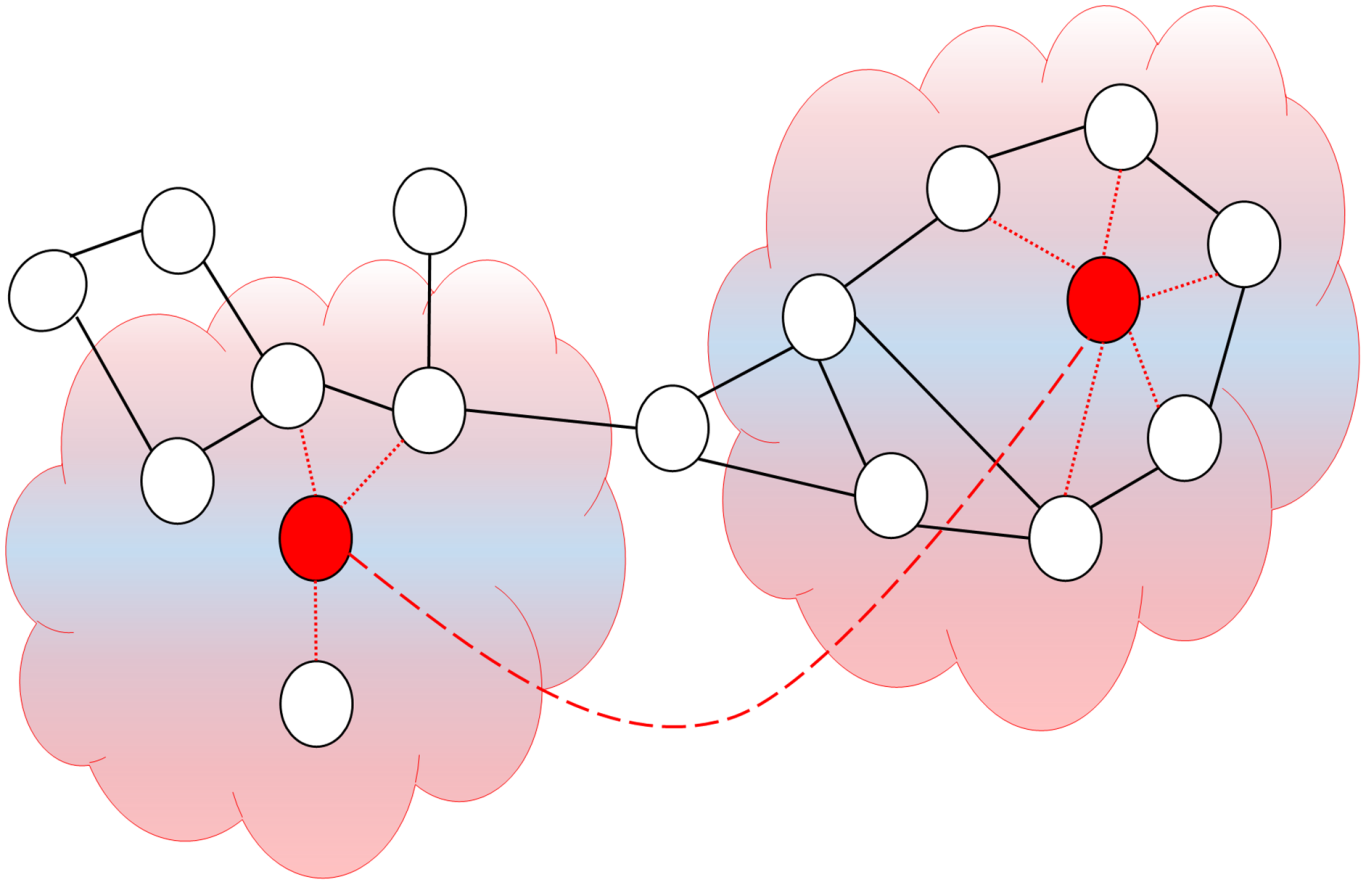
Simulation of wormhole attack.

In a wormhole attack, the attacker connects two network nodes physically separated from one another using a quick communication path called a wormhole tunnel as can be seen in Fig. 2. This communication platform can be an Ethernet cable, high-speed wireless communication, or fiber optic communication. When the wormhole tunnel is implemented, the attacker captures the packets directed by the nodes on one side of the network and spreads them through the wormhole tunnel on the other side. The wormhole nodes behave completely transparently, making them invisible to the network; therefore, it is operational even without network IDs or cryptographic keys (Ma et al., 2017). Table 1 displays the comparative analysis of currently available wormhole attack detections in literature.

**Table 1:** Wormhole attack detections in literature.

Citation	Detection method	Performance evaluation metric	Strengths
(Alajlan, 2022)	Location information	*False Detection Rate (FDR)	*Detection in completely distributed network.
			*Detects and prevents the simplex and duplex wormhole attacks.
		*Detection Time	
(Pawar & Jagadeesan, 2021)	Deep learning	True Detection Rate (TDR)	*Improves the detection probability compared to conventional methods.
(Vaniprabha & Poongodi, 2019)	*Elliptic Curve Cryptography (ECC)	*False Positive Rate (FPR)	*Enhances data accuracy of the collected data with minimized delay.
			*97% packet delivery ratio
			*0.8 ms packet dropping
			*2.4 ms for key generation
		*Packet Delivery Ratio (PDR)	*0.96 ms for secret key exchange.
	*Transmission model		
(Li & Wang, 2019)	*Distance Vector Hop (DV Hop) Algorithm	Localization Error	*Localization error of 112.3%, 10.2%, 41.7%, 6.9% reduction
	*Neighbours Based		
(Padmanabhan & Manickavasagam, 2018)	Sequential probability ratio test	*Resource Consumption	*Detection is faster with increasing mobility.
		*Computation Overhead	
		*Detection Accuracy	
		*True Detection Rate (TDR)	
			*System is highly customizable.
		*Communication Overhead	
			*No additional resource requirement.
(Patel, Aggarwal & Chaubey, 2018)	Neighbours Based	*Computation Overhead	*Does not require any additional hardware.
		*Detection Accuracy	
(Singh et al., 2018)	*Behaviour Based	Efficiency	*Low rate of the infectious node for different Communication radius.
	*Stability theory of differential equations		
(Shu et al., 2017)	Quantum Ant Colony Algorithm	Energy Consumption	*For large scale WSN routing.
(Wang et al., 2017)	Microscopic mathematical model	*Computational Cost	*Considers diffusion with a mobile worm carrier.
			*Minimize cost by 50% compared to others.
		*Detection Accuracy	
(Kumar et al., 2016)	*Signature Based	*Throughput	*Improves the data reliability
	*Rule base	*Packet Delivery Ratio (PDR)	
(Manikandan, Satyaprasad & Rajasekhararao, 2016)	Round Trip Time (RTT) base	*Efficiency	*Higher efficiency and Throughput with cost effective.
		*Computational Cost	
		*Throughput	
(Singh, Singh & Singh, 2016b)	*Rank Based	*Resource Consumption	*Capacity to defend against almost all categories of wormhole attacks without depending on any required additional hardware
	*Watchdog		
	*Delphi scheme		
		*Computational Cost	
(Mukherjee et al., 2016)	*Range Based	*Efficiency	*It can detect both short path and long path wormhole links.
	*Neighbours Based		
(Lai, 2016)	RPL based	Efficiency	*Can identify wormholes effectively under various WSN

### Sybil attacks

**Figure 3:**
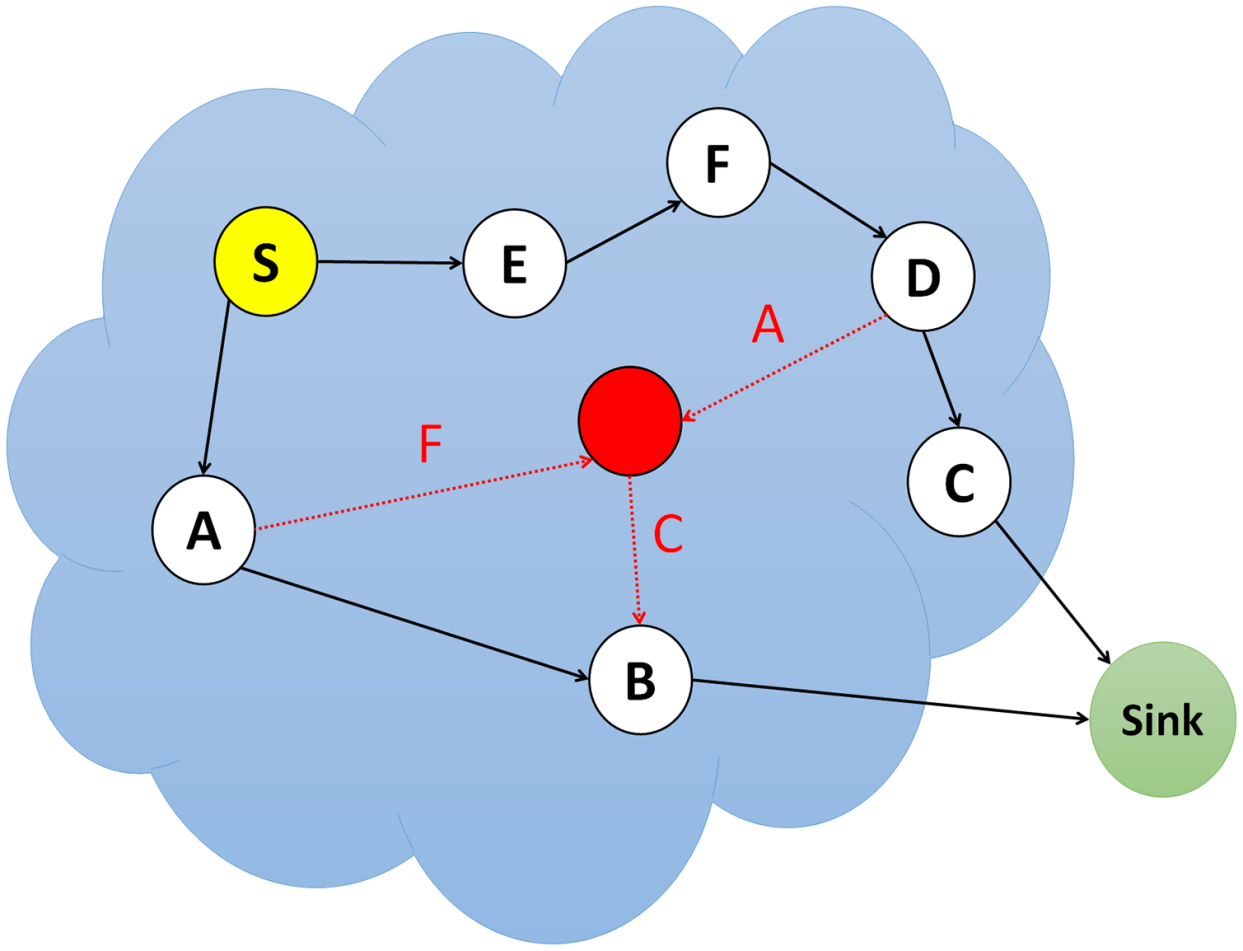
Simulation of sybil attack.

The 1973 book “Sybil” is about Shirley Mason, a mental health patient with multiple false identities (Schreiber, 1973). Sybil was named after the WSN nodes with false identities for this rationale. The most vulnerable peer-to-peer networks to Sybil attacks are distributed networks. In its most basic form, Fig. 3 shows how a malicious node uses the Sybil attack to place itself in a position of multiple other nodes. Sybil’s attack can stop redundancy in distributed networks by falsifying other nodes’ identities and preventing accurate distribution.

Each identity should be linked to a physical node to defend against Sybil attacks. There are two ways to achieve the stated objective. The first method is direct acknowledgment, in which the node checks the accuracy of its interacting node directly. A second method is a form of indirect acknowledgment in which the verified node accepts or rejects the other node. The following are three ways to detect a Sybil attack:

- Evaluating the radio source
- Determining the critical correctness for pre-distributed keys
- Node Registration and Location discovery.

Table 2 displays the comparative analysis of currently available Sybil attack detections in literature.

**Table 2:** Sybil attack detections in literature.

Citation	Detection method	Performance evaluation metric	Strengths
(Saravanakumar et al., 2022)	*Encryption Method	*Packet Loss Ratio (PLR)	*Achieve higher data delivery with a minimum delay
		*Computation Overhead	
	*Artificial deep neural networks	*Throughput	
		*End-to-End Delay	
(Singh & Saini, 2021b)	*Received Signal Strength (RSS)	*Energy Consumption	*Utilized for the usage of energy consumption, effectiveness of detecting Sybil attacks inside clusters.
	*PCTBC: Power Control Tree Based Cluster Approach		
(Angappan et al., 2021)	*Received Signal Strength (RSS)	*True Detection Rate (TDR)	*Highly efficient in detection ratio, energy utilisation, memory usage, computation, and Communication requirement
		*Communication Overhead	
	*Localization Based	*Energy Consumption	
		*Computation Overhead	
	*Cluster based		
		*Memory Overhead	
(Raghav, Thirugnansambandam & Anguraj, 2020)	Bee algorithm	*Efficiency	*Better data efficiency with security
(Dong, Zhang & Zhou, 2020)	Distance Vector Hop (DV Hop) Algorithm	*Localization Error	*Improve the security of the node localization in WSN.
			*Reduces the average localization error by 3% than the traditional DV Hop.
(Wang & Feng, 2020)	Received Signal Strength (RSS)	*True Detection Rate (TDR)	*Resolve time difference
(Jamshidi et al., 2019a)	*Learning Automaton (LA)	*True Detection Rate (TDR)	*Detects 100% of Sybil nodes
		*Communication Overhead	
		*False Detection Rate (FDR)	
		*Computation Overhead	
	*Client puzzles theory		*5% false detection rate
(Jamshidi et al., 2019c)	Received Signal Strength (RSS)	*True Detection Rate (TDR)	*Detect 99.8% of Sybil nodes
		*Communication Overhead	
			*0.008% false detection rate
		*False Detection Rate (FDR)	
			*True detection rate
(Jamshidi et al., 2019b)	Information based	*True Detection Rate (TDR)	*Detect 99% of Sybil nodes
			*5% false detection rate
		*False Detection Rate (FDR)	
(Li & Cheffena, 2019)	*Multi Kernel Based Expectation Maximization (MKEM)	*Detection Accuracy	*High accuracy on detecting Sybil
	*Gap statistical analysis method		
			*Can guarantee the detection accuracy even if the number of Sybil attackers increases.
	*Kernel parameter optimization		
(Vaniprabha & Poongodi, 2019)	*Elliptic Curve Cryptography (ECC)	*False Positive Rate (FPR)	*Enhances data accuracy of the collected data with minimized delay.
			*97% packet delivery ratio
			*0.8 ms packet dropping
			*2.4 ms for key generation
		*Packet delivery Ratio (PDR)	*0.96 ms for secret key exchange.
	*Transmission model		
(Shehni et al., 2018)	Watchdog	*True Detection Rate (TDR)	*Low extra Communication overhead
		*Communication Overhead	*Detection measures of performance *True
			Detection Rate (TDR)
		*False Detection Rate (FDR)	*False Detection Rate (FDR)
		*Network Performance	
(Yuan et al., 2018)	*Received Signal Strength (RSS)	*True Detection Rate (TDR)	*Requires minimal overhead
			*Works well based on the received signal strength
			*Effective in detecting and defending against sybil attacks
		*Communication Overhead	
	*Localization Based		*High detection rate
(Wang, Wen & Zhao, 2018)	*Deep learning	*Detection Accuracy	*94.39% classification accuracy
	*Stacked Denoising Autoencoder (SDA)		
	*Back propagation algorithm		
	*Complex network theory		*More robust and efficient even in the existent of huge baneful beacons.
(Jamshidi et al., 2017)	Behaviour Based	*True Detection Rate (TDR)	*Identify 94% of Sybil nodes
		*False Detection Rate (FDR)	
			*False detection rate
(Li et al., 2017)	Localization Based	*Localization Error	*Superior in terms of malicious nodes identification and performance improvement.
(Razaque & Rizvi, 2017)	Data Aggregation	*Detection Accuracy	*Prevent and detect both sinkhole and Sybil attacks in the presence of static and mobile sensor nodes
		*Communication Overhead	
		*Energy Consumption	
(Raja & Beno, 2017)	Security mechanism and Fujisaki Okamoto (FO) algorithm	*Throughput	*Increase the performance of the network.
		*Energy Consumption	
		*Packet Delivery Ratio (PDR)	
(Alsaedi et al., 2017)	Energy Trust System (ETS)	*True Detection Rate (TDR)	*Robust in detecting sybil attacks in terms of the true and false positive rates. *70% detection at the first level, which significantly increases to 100% detection at the second level *Reduces communication overhead, memory overhead, and energy consumption
		*Energy Consumption *Communication Overhead *Memory Overhead *False Positive Rate (FPR) *Resource Consumption	
(Khan & Khan, 2016)	Signed response (SRES) authentication	*Power Consumption	*Lesser computational cost
		*Computational Cost	*Power consumption
(Singh, Singh & Singh, 2016a)	Trust Based	*True Detection Rate (TDR)	*Significant attack detection rate
(Kumar et al., 2016)	*Signature Based	*Throughput	*Improves the data reliability
		*Packet delivery Ratio (PDR)	
	*Rule base		
(Vamsi & Kant, 2016)	Neighbour Based	*False Positive Rate (FPR)	*Robust in detecting Sybil attacks with very low false positive and false negative rates.
		*False Negative Rate (FNR)	
(Saleem et al., 2016)	Encryption Based	*Resource Consumption	*Efficiently protect WSNs Sybil attacks.
		*Energy Consumption	
		*Computation Overhead	
		*Packet Delivery Ratio (PDR)	
		*Computational Cost	

### Grayhole/Selective forwarding (SF) attacks

**Figure 4:**
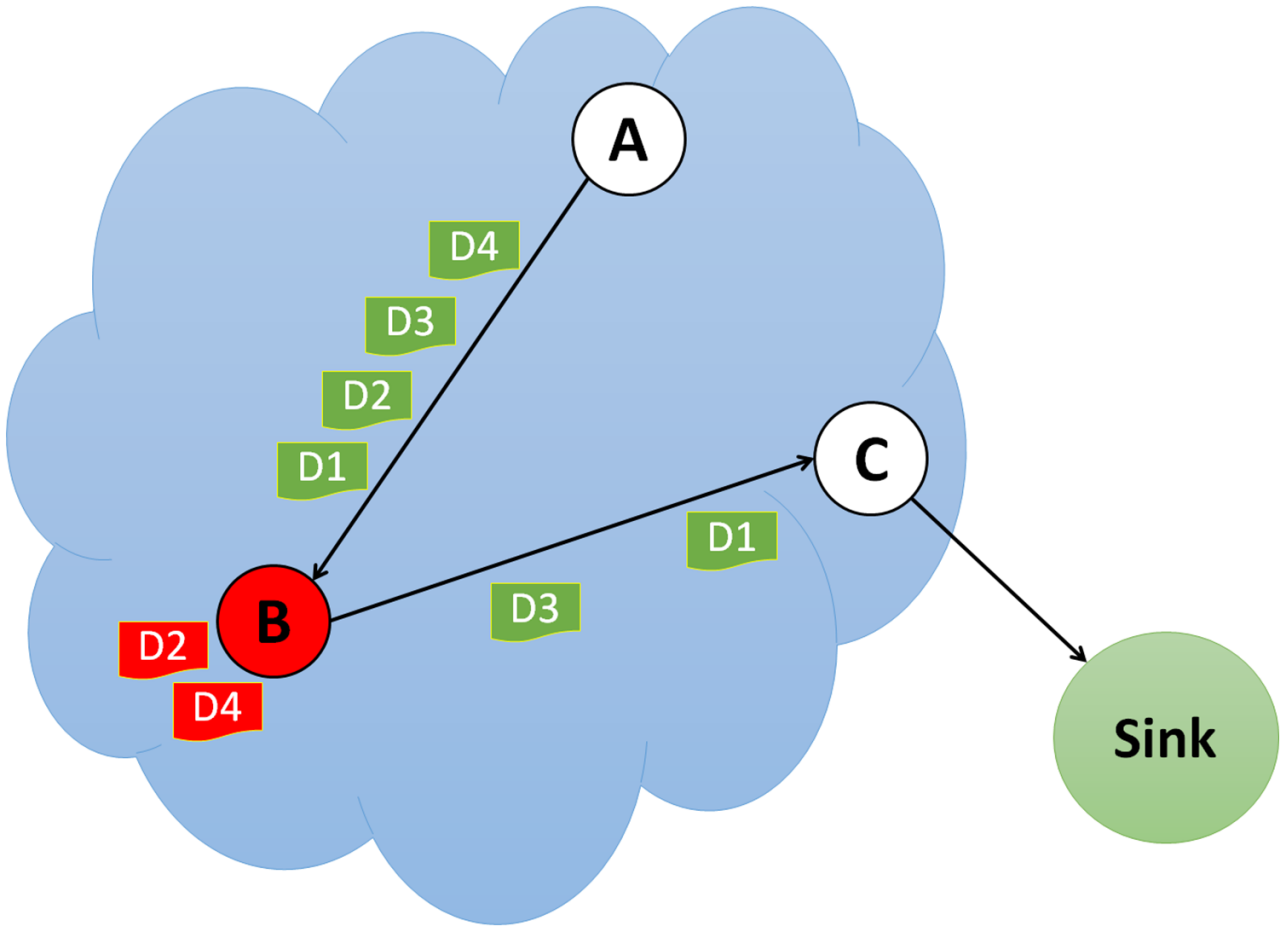
Simulation of grayhole/selective forwarding (SF) attack.

Multi-step routing networks forward packets safely and unchanged to the parent node. Attacker nodes employing grayhole/selective forwarding may decide not to forward or drop specific packets or alter them before forwarding. A standard grayhole/selective forwarding attack is that the attacker node avoids sending any packet to the next node and deletes them. As shown in Fig. 4, if an attacker node feels threatened by its neighbors, it explores a different path and decides to leave the current path (La, Fuentes & Cavalli, 2016).

Since the attack is conducted through authenticated nodes, the authentication mechanisms must be improved to detect and prevent grayhole/selective forwarding attacks. So far, several solutions have been proposed to deal with these types of attacks, such as:

- Attack identification through the concept of node authentication.
- The concept of a multithreaded data stream.
- Detection using the heterogeneous network theory.

Table 3 displays the comparative analysis of currently available Grayhole/Selective Forwarding (SF) attack detections in literature.

**Table 3:** Grayhole/Selective forwarding (SF) attack detections in literature.

Citation	Detection MEthod	Performance evaluation metric	Strengths
(Chinnaraju & Nithyanandam, 2022)	*Neighbour Based	*Packet Delivery Ratio (PDR)	Instead of blocking the entire host, it specifically eliminates the malicious nodes.
	*Threshold Based	*Network Performance	
(Liu & Wu, 2021)	*Cluster based	*False Detection Rate (FDR)	*Low False Detection Rate (FDR) of 1%
	*Voting decision method	*Energy Consumption	*Low Missed Detection Rate (MDR) of 5%
		*Missed Detection Rate (MDR)	*Negligible energy consumption
(Singh & Saini, 2021a)	Learning based	*Data Transmission Rate (DTR)	Better data transmission
		*Efficiency	
(Lal & Prathap, 2021)	Provenance based technique	*Throughput	Assuring data trustworthiness
(Derhab et al., 2020)	*Neighbour Based	*Detection Accuracy	Defending against upstream node attack
	*Upstream node effect	*Energy Consumption	
(Fang et al., 2020)	Trust Based	*Data Transmission Rate (DTR)	*Prevent the appearance of network holes
		*Energy Consumption	*Balance the network load
			*Promote the survivability of the network.
(Fu et al., 2020)	Data Clustering Algorithm (DCA)	*False Detection Rate (FDR)	*Low missed detection rate of 1.04%
		*Energy Consumption	*False detection rate of 0.42%
		*Missed Detection Rate (MDR)	*Low energy consumption.
(Alqahtani et al., 2019)	*Genetic algorithm	*True Detection Rate (TDR)	*High detection rates of 98.2%, 92.9%, 98.9%, and 99.5% for flooding, scheduling, grayhole, and blackhole attacks
	*Extreme Gradient Boosting (XGBoot) classifier		*99.9% for normal traffic
(Devi & Ganesan, 2019)	*Trust Based	*Packet Loss Ratio (PLR)	Pay attention to consecutive packet dropping.
	*Watchdog		
(Mehetre, Roslin & Wagh, 2019)	*Trust Based	*Network Lifetime	Assurance to prolong the network lifespan and the probability of secure routing path in the network.
	*Cuckoo search algorithm	*Network Performance	
(Zhang & Zhang, 2019)	Watchdog	*False Alarm Rate	*Reduces the false detection rate by 25%
		*False Detection Rate (FDR)	*Improves the detection accuracy by 10%
		*Detection Accuracy	*Energy consumption
		*Energy Consumption	
(Kaur et al., 2018)	Threshold Based	*Packet Loss Ratio (PLR)	Improvement in terms of dead nodes, Throughput, and packet loss.
		*Throughput	
(Terence & Purushothaman, 2019)	Behaviour Based	*End-to-End Delay	*Improve packet delivery ratio, Throughput, and end to end delay
		*Throughput	*Lesser false positive (less than 6%)
		*False Positive Rate (FPR)	*False negative (less than 4%)
		*Packet Delivery Ratio (PDR)	
		*False Negative Rate (FNR)	
(Jararwah, 2018)	*Watchdog	*Detection Accuracy	*Any sensor drops more than 20% is considered malicious.
	*Fog Computing	*Power Consumption	
(Yi et al., 2018)	*Trust Based	*Efficiency	*Safety, filtering efficiency, and data availability.
	*Signature Based		
(Pu & Lim, 2018)	*Timeout and hop by hop retransmission techniques	*Packet Loss Ratio (PLR)	Improve the detection rate and Packet Delivery Ratio (PDR) as well as reduce the energy consumption, false detection rate, and successful drop rate.
	*Behaviour Based	*False Detection Rate (FDR)	
		*Packet Delivery Ratio (PDR)	
		*True Detection Rate (TDR)	
		*Energy Consumption	
(Zhu et al., 2018)	*Adaptive learning automata and Communication quality	*Communication Overhead	*Low communication overhead
	*Neighbour Based		
	*Behaviour Based		
(Ji, 2018)	Threshold Based	*Data Transmission Rate (DTR)	Saves Communication resource
		*Resource Consumption	
		*Communication Overhead	
(Farooqi & Khan, 2017)	Cloud based	*Throughput	Favour LEACH++ over LEACH under attack with respect to Throughput and energy consumption.
		*Energy Consumption	
(Garcia-Font, Garrigues & Rifa-Pous, 2017)	Classification schema	*Efficiency	Demonstrate the use of the classification schema
(Gara, Ben Saad & Ben Ayed, 2017)	IPv6 routing protocol for low power and lossy networks (6LowPAN)	*True Detection Rate (TDR)	The detection probability is 100% for selective attackers under some cases.
(Elhoseny et al., 2016)	*Elliptic curve cryptography algorithm	*Network Lifetime	*Much improved network lifetime
	*Encryption method	*Energy Consumption	*Reduced the energy consumption
(Mathur, Newe & Rao, 2016)	*Neighbour Based	*Detection Accuracy	*96% accuracy for SF attacks
	*Threshold Based	*True Detection Rate (TDR)	*83% accuracy for malicious node
	*Cryptography		
(Saleem et al., 2016)	Encryption Based	*Resource Consumption	Efficiently protect WSNs from selective forwarding, spoofing, replay, Hello flood, and Sybil attacks
		*Computational Cost	
		*Computation Overhead	
		*Packet Delivery Ratio (PDR)	
		*Energy Consumption	
(Liu et al., 2016)	Multi Data and Multi-ACK (MDMA scheme)	*Data Transmission Rate (DTR)	*Success rate of data transmission,
		*Network Lifetime	*Detecting SFA
			*Identifying malicious nodes
(Zhou et al., 2016)	Cluster based	*False Alarm Rate	*False alarm rate is lowered by 25.7% *Network lifespan is prolonged by 54.84%
		*Network Lifetime	
		*Detection Accuracy	
(Ren et al., 2016)	Behaviour Based	*Detection Accuracy	*Accurately detect SF attacks
		*Packet Delivery Ratio (PDR)	*Improve the data delivery ratio

### Blackhole attack

**Figure 5:**
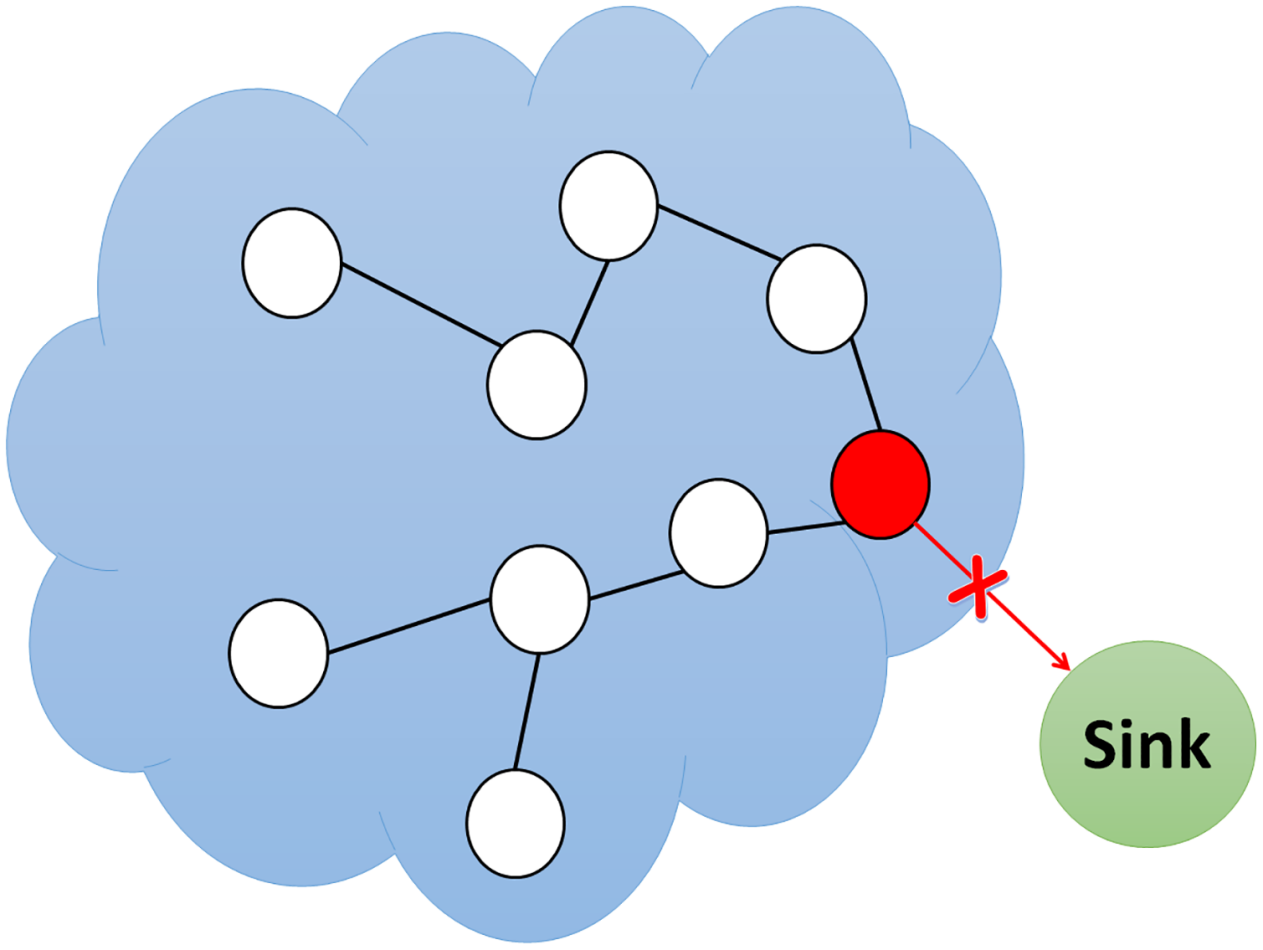
Simulation of blackhole attack.

In a blackhole attack, the attacker pulls traffic to the network by broadcasting fake routing information to find the shortest path. This malicious node pretends to have the shortest path while sending fake messages. As a result, the source node ignores the routing table and utilizes this node to send packets. The blackhole node then begins to drop the sent packets, as shown in Fig. 5, causing a network service interruption or provision.

The network may suffer severe damage because of a blackhole attack; however, neighboring nodes can quickly identify the malicious nodes by keeping an eye on their activity. A risky fake route will be proposed that will not deliver the packets to their intended location if the malicious node responds to the request message before the valid node does. The malicious node will disrupt the network and drop the packets, disrupting packet movement in the network. Table 4 displays the comparative analysis of currently available blackhole attack detections in literature.

**Table 4:** Blackhole attack detections in literature.

Citation	Detection method	Performance evaluation metric	Strengths
(Saravanakumar et al., 2022)	*Encryption method	*Packet Loss Ratio (PLR)	*Achieve higher data delivery with a minimum delay
	*Artificial deep neural networks	*Computation Overhead	
		*Throughput	
		*End-to-End Delay	
(Pawar & Jagadeesan, 2021)	Deep learning	True Detection Rate (TDR)	*Improves the detection probability compared to conventional methods.
(Karakoç & Çeken, 2021)	*Blockchain	*True Detection Rate (TDR)	Promising solution for securing SDN enabled WSN structures.
	*Signature Based		
(Nosratian, Moradkhani & Tavakoli, 2021)	*Fuzzy Based	*Computational Cost	Best response for path selection with the least cost for the target performance
	*Data mining		
	*Genetic Algorithm (GA)		
	*Teaching Learning-Based Optimization (TLBO)		
(Yadav & Mishra, 2020)	Blockchain	*End-to-End Delay	Successful identification and occurrence of malicious nodes, end to end delay, packet delivery ratio and throughput
		*Packet Delivery Ratio (PDR)	
		*Throughput	
(Alqahtani et al., 2019)	*Genetic algorithm	*True Detection Rate (TDR)	*High detection rates of 98.2%, 92.9%, 98.9%, and 99.5% for flooding, scheduling, grayhole, and blackhole attacks
	*Extreme Gradient Boosting (XGBoot) classifier		*99.9% for normal traffic
(Terence & Purushothaman, 2019)	Behaviour Based	*End-to-End Delay	*Improve packet delivery ratio, Throughput, and end to end delay
		*Throughput	*Lesser false positive (less than 6%)
		*False Positive Rate (FPR)	*False negative (less than 4%)
		*Packet Delivery Ratio (PDR)	
		*False Negative Rate (FNR)	
(Mehetre, Roslin & Wagh, 2019)	*Trust Based	*Network Lifetime	Assurance to prolong the network lifespan and the probability of secure routing path in the network.
	*Cuckoo search algorithm	*Network Performance	
(Sunder & Shanmugam, 2019)	Jensen Shannon Divergence Based Independent Component Analysis (JDICA) technique	*False Alarm Rate	*Greater accuracy
		*End-to-End Delay	*Increasing the packet delivery ratio
		*Detection Accuracy	*Reducing delay
		*Packet Delivery Ratio (PDR)	
		*True Detection Rate (TDR)	
		*Energy Consumption	
		*Detection Time	
(Bilgin & Baktir, 2019)	*Symmetric key cryptographic algorithm	*End-to-End Delay	*Same delivery ratios as the original *ad hoc* on demand distance vector routing protocol
	*Advanced Encryption Standard (AES)	*Packet Delivery Ratio (PDR)	*Does not cause extra Communication delay.
	*Signature Based		
(Ariffin, Mokhtar & Abd Rahman, 2018)	Network performance	*Network Lifetime	Guidance for other research for improving the security of other protocol
		*Energy Consumption	
		*Packet Delivery Ratio (PDR)	
(Farooqi & Khan, 2017)	Cloud based	*Throughput	Favour LEACH++ over LEACH under attack with respect to Throughput and energy consumption.
		*Energy Consumption	
(Almon, Riecker & Hollick, 2017)	Behaviour Based	*Localization Error	Fully localized intrusion detection system requiring no collaboration.
		*Data Transmission Rate (DTR)	
		*Network Performance	
(Wazid & Das, 2017)	Group based technique	*False Positive Rate (FPR)	*90% detection rate
		*True Detection Rate (TDR)	*3.75% false positive rate
(Kaur, Jain & Chaba, 2017)	Network performance	*Network Performance	*Malicious node is successfully detected
			*Prevents the deterioration in the performance of WSN
(Aljumah & Ahanger, 2017)	Futuristic method	*Network Performance	Detect and prevent the blackhole attack in WSNs
(Kumar et al., 2016)	*Signature Based	*Throughput	*Improves the data reliability
	*Rule base	*Packet Delivery Ratio (PDR)	
(Manikandan, Satyaprasad & Rajasekhararao, 2016)	Round Trip Time (RTT) base	*Efficiency	*Higher efficiency and Throughput with cost effective.
		*Computational Cost	
		*Throughput	
(Mathur, Newe & Rao, 2016)	*Neighbour Based	*Detection Accuracy	*96% accuracy for SF attacks
	*Threshold Based	*True Detection Rate (TDR)	*83% accuracy for malicious node
	*Cryptography		
(Wazid & Das, 2016)	Cluster based	*Network Lifetime	*98.6% detection rate
		*False Positive Rate (FPR)	*1.2% false positive rate
		*True Detection Rate (TDR)	

### Sinkhole attacks

**Figure 6:**
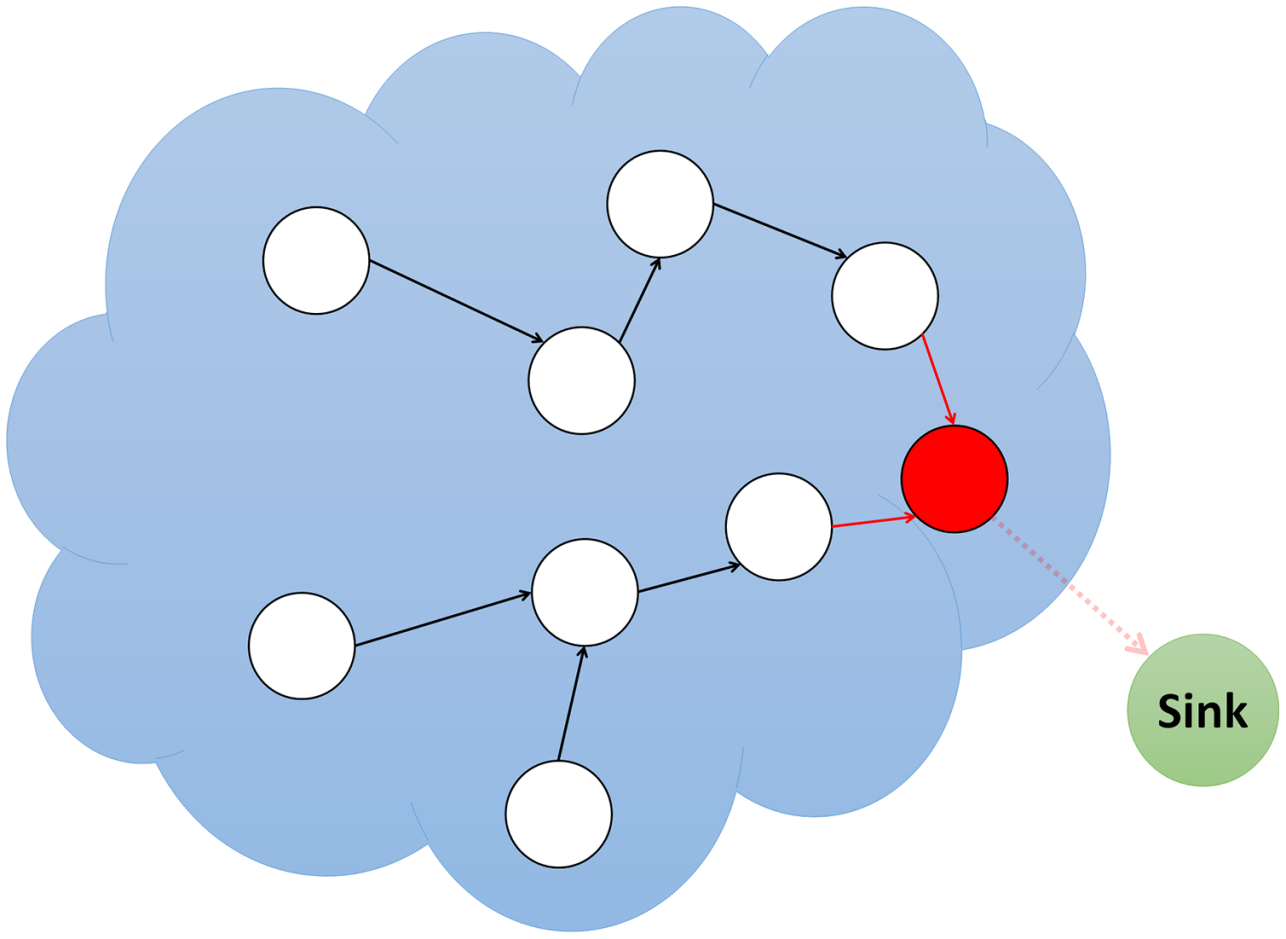
Simulation of sinkhole attack.

Sinkhole attacks are one of the most appealing but dangerous attacks in WSNs. This attack is notable for its ability to launch another attack in the middle of the attack. The sinkhole attack uses a node with false information and specifications to fool neighboring nodes into sending their data to the attacker node. At this time, the attacker can apply any changes to the information, including changing the packet, rejecting packets, or utilizing other attacks. Figure 6 illustrates a simulation of a sinkhole attack.

In WSNs, communication is hop-to-hop, meaning the packet is conveyed from one node to another to reach the destination. In this case, the nodes usually choose a path that has a lower hop and selects a node as its parent, which is in a less hop count path, known as an optimal path. The attack starts when a sensor node decides to show itself as desirable to other nodes (Isidro & Ashour, 2021). Because of optimal path selection in WSNs, nodes try to select the best path, which also has the least cost to transmit their packets. The cost may include several factors such as processing, energy consumed, distance and load. Therefore, a malicious node in the sink attack somehow shows itself to its neighbors that they think it has the lowest cost and the shortest path to the sink. In this case, the attack enters its primary phase, as neighboring nodes select the malicious node as their parent and send information to it by lack of knowledge that the node will announce fake and false information about its distance to the base station entirely unrealistic. At this time, a penetration range is created in the network, massive network traffic comes to this node, and much information gets changed or forged. The malicious node can be a laptop-class type with several process power and energy and can continue to sabotage for a long time (Reji et al., 2017). Table 5 displays the comparative analysis of currently available sinkhole attack detections in literature.

**Table 5:** Sinkhole attack detections in literature.

Citation	Detection method	Performance evaluation metric	Strengths
(Nithiyanandam & Parthiban, 2020)	*Artificial Bee Colony algorithm	*Network Lifetime	Impact of the proposed algorithm on WSN scenario when compared to other existing algorithms.
	*Voting based	*Energy Consumption	
(Sejaphala & Velempini, 2020)	Hop Count Based	*Packet Loss Ratio (PLR)	Performance of the network
		*Packet Delivery Ratio (PDR)	
(Yuan & Wang, 2020)	*Signature Based	*Energy Consumption	*Occupies a small amount of key storage space
	*Double encryption method		*Prevents attackers from initiating sinkhole attacks and resend attacks, thereby enhancing network security.
	*Identity-Based Encryption (IBE) algorithm		
	*Elliptic Curve Cryptography (ECC)		
(Terence & Purushothaman, 2019)	Behaviour Based	*End-to-End Delay	*Improve packet delivery ratio, Throughput, and end to end delay
		*Throughput	*Lesser false positive (less than 6%)
		*False Positive Rate (FPR)	*False negative (less than 4%)
		*Packet Delivery Ratio (PDR)	
		*False Negative Rate (FNR)	
(Vaniprabha & Poongodi, 2019)	*Elliptic Curve Cryptography (ECC)	*False Positive Rate (FPR)	*Enhances data accuracy of the collected data with minimized delay.
	*Transmission model	*Packet Delivery Ratio (PDR)	*97% packet delivery ratio
			*0.8 ms packet dropping
			*2.4 ms for key generation
			*0.96 ms for secret key exchange.
(Zhang et al., 2019)	Hop Count Based	*False Positive Rate (FPR)	*Detection rate increased about by 30%
		*Energy Consumption	*False positive rate decreased about by 25%
		*True Detection Rate (TDR)	*Obtain a high energy saving
(Shang et al., 2019)	*Link quality	*Network Performance	*Can detect not only Sinkhole and DoS attacks, but also other specific vulnerabilities
	*Evidence theory		*Better performance
(Farooqi & Khan, 2017)	Cloud based	*Throughput	Favour LEACH++ over LEACH under attack with respect to Throughput and energy consumption.
		*Energy Consumption	
(Raja & Beno, 2017)	Security mechanism and Fujisaki Okamoto (FO) algorithm	*Throughput	*Increase the performance of the network.
		*Energy Consumption	
		*Packet Delivery Ratio (PDR)	
(Reji et al., 2017)	Network performance	*Computation Overhead	Variation of the parameters in the attack scenarios
		*Energy Consumption	
(Jahandoust & Ghassemi, 2017)	*Subjective logic and probabilistic extension of timed automata	*Packet Loss Ratio (PLR)	*Low packet loss
	*Behaviour Based	*False Negative Rate (FNR)	*False positive and false negative results reduce
		*False Positive Rate (FPR)	
(Wazid et al., 2016)	Cluster based	*Computation Overhead	*95% detection rate
		*Communication Overhead	*1.25% false positive rate
		*True Detection Rate (TDR)	*Computation and communication efficiency
		*False Positive Rate (FPR)	
(Keerthana & Padmavathi, 2016)	Enhanced Particle Swarm Optimization Technique	*False Alarm Rate	*Detection rate, False Alarm rate, Packet delivery ration, Message drop and Average delay
		*True Detection Rate (TDR)	
		*Packet Delivery Ratio (PDR)	
		*End-to-End Delay	
		*Packet Loss Ratio (PLR)	

### Replay attack

**Figure 7:**
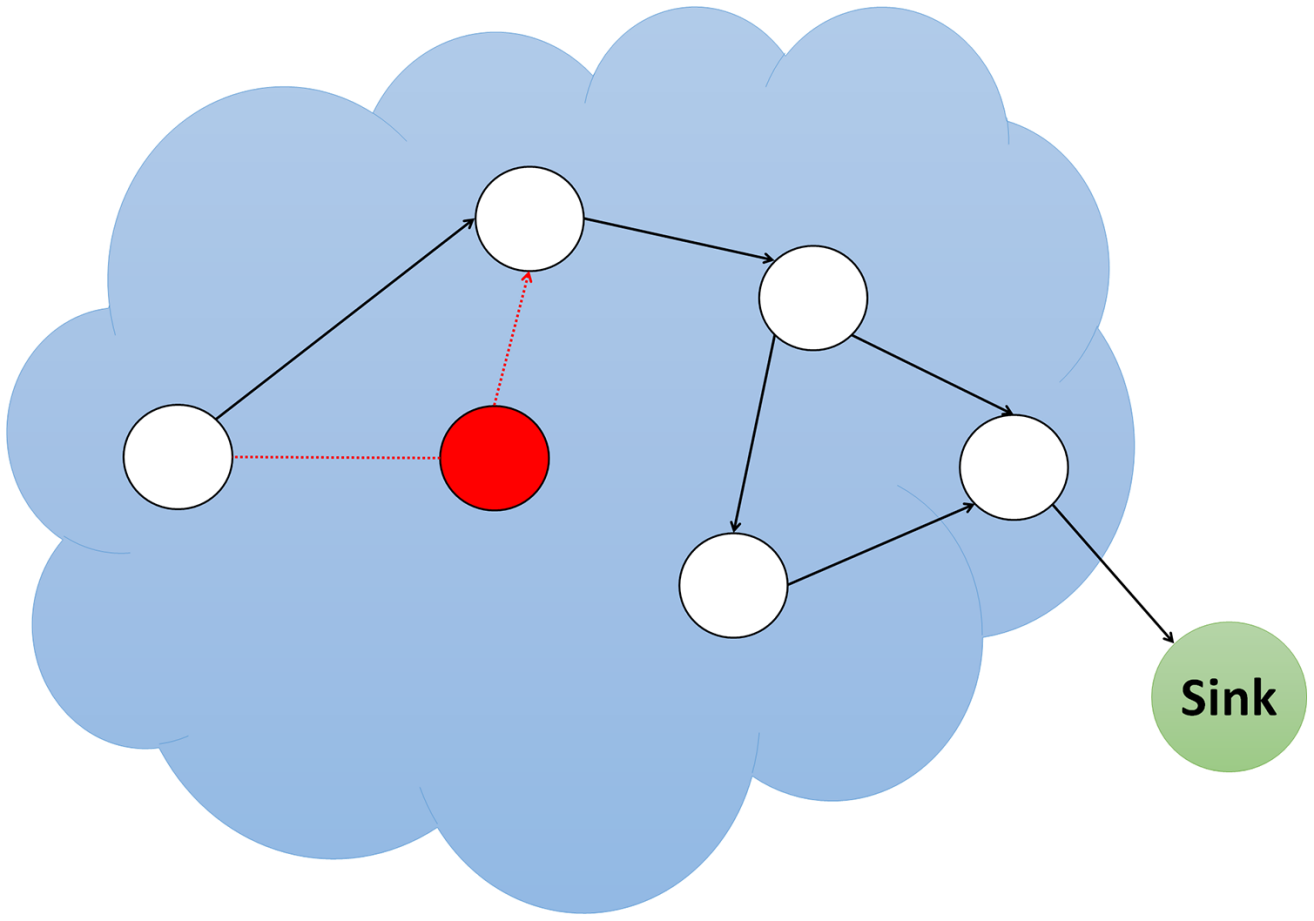
Simulation of replay attack.

The furthermost standard direct attack in contrast to a routing protocol is to target the routing data between the nodes. Unprotected routing in WSNs causes such vulnerabilities on routing because each node in the WSNs can perform as a router, and thus can promptly affect routing data (Chaki & Ashour, 2021). By replay attack, intruders can cause routing loops, wrong error packets, network division, increase end-to-end latency, and increase or shorten the path. Figure 7 displays a simulation of replay attack.

The Code Verification Identity Packet can be used to deal with replay attacks, which are attached to the original packet. The recipient can identify the fake or modified packet by adding a packet confirming the code’s identity. Furthermore, counters and timestamps can be used in the packet being sent to counteract the repetition of routing information. In general, a solution to such attacks can be found by validating nodes and encoding data packets (Pathan, 2016). Table 6 displays the comparative analysis of currently available replay attack detections in literature.

**Table 6:** Replay attack detections in literature.

Citation	Detection method	Performance evaluation metric	Strengths
(Karakoç & Çeken, 2021)	*Blockchain	*True Detection Rate (TDR)	Promising solution for securing SDN enabled WSN structures.
	*Signature Based		
(Bilgin & Baktir, 2019)	*Symmetric key cryptographic algorithm	*End-to-End Delay	*Same delivery ratios as the original *ad hoc* on demand distance vector routing protocol
	*Advanced Encryption Standard (AES)	*Packet Delivery Ratio (PDR)	*Does not cause extra communication delay.
	*Signature Based		
(Wang, Wen & Zhao, 2018)	*Deep learning	*Detection Accuracy	*94.39% classification accuracy
	*Stacked Denoising Autoencoder (SDA)		*More robust and efficient even in the existent of huge baneful beacons.
	*Back propagation algorithm		
	*Complex network theory		
(Wen & Wang, 2018)	Trust Based	Communication Overhead	*Communication load
(Saleem et al., 2016)	Encryption Based	*Resource Consumption	*Efficiently protect WSNs Sybil attacks.
		*Energy Consumption	
		*Computation Overhead	
		*Packet Delivery Ratio (PDR)	
		*Computational Cost	
(Garcia-Otero & Poblacion-Hernandez, 2016)	*Received Signal Strength (RSS)	True Detection Rate (TDR)	*Does not require any calibration process
	*Location information		*Robust to changing environmental conditions

### Spoofing attacks

**Figure 8:**
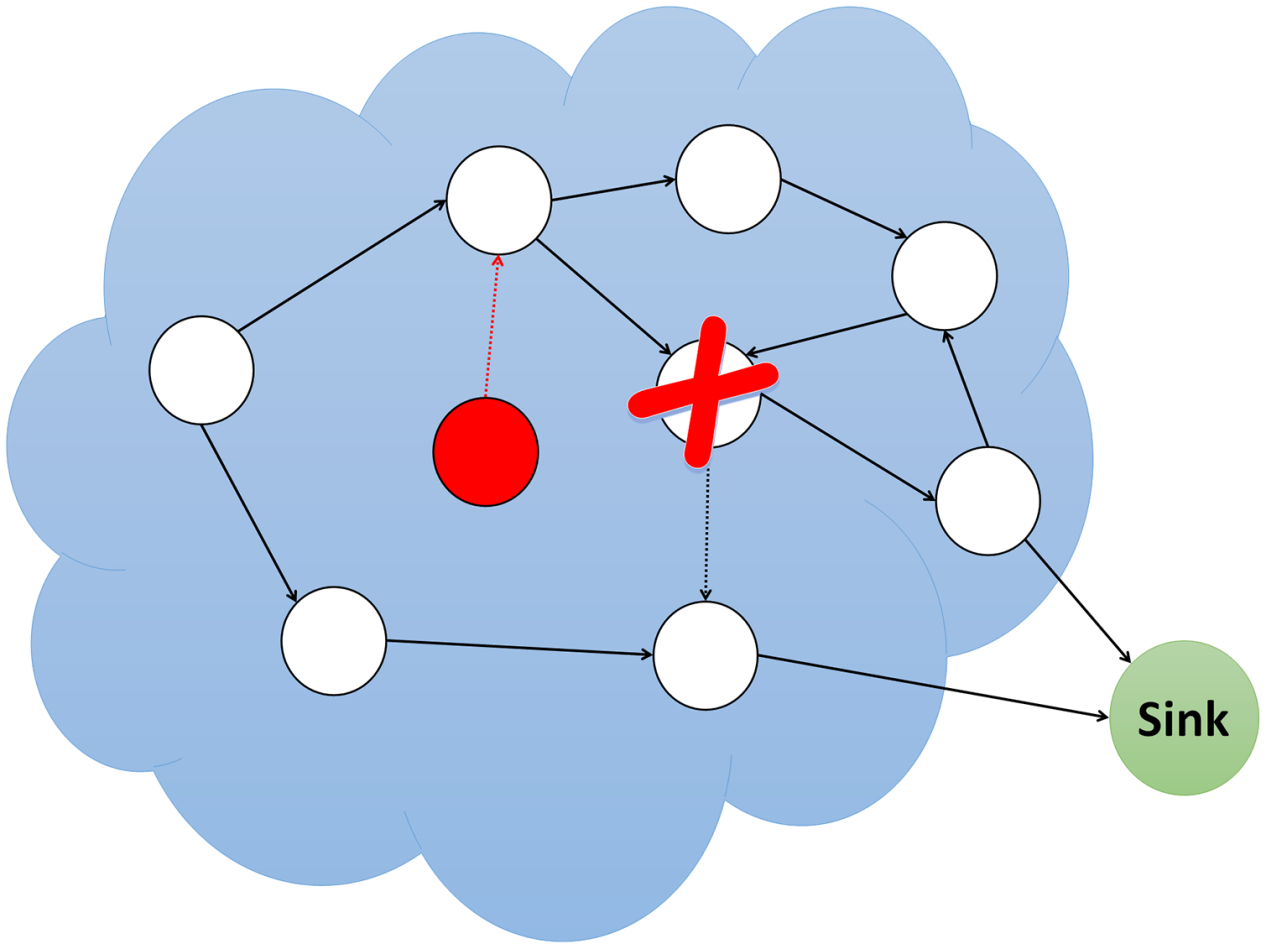
Simulation of spoofing attack.

Spoofing is a direct and standard attack against the routing protocol. This attack aims to obtain the path of information exchange between two nodes. Attackers will be able to create routing loops, attract or decline network traffic, extend, or shorten resource paths, generate false error messages, segment the network, and ultimately increase end-to-end traffic (Huan, Kim & Zhang, 2021). Figure 8 illustrates a simulation of a spoofing attack.

The common solution for this type of attack is authentication and validation. Table 7 displays the comparative analysis of currently available spoofing attack detections in literature.

**Table 7:** Spoofing attack detections in literature.

Citation	Detection method	Performance evaluation metric	Strengths
(Huan, Kim & Zhang, 2021)	Reverse time synchronization framework	Detection Accuracy	Both centralized and distributed NISA could provide accurate node identification and Spoofing attack detection.
(Raghav, Thirugnansambandam & Anguraj, 2020)	Bee algorithm	*Efficiency	*Better data efficiency with security
(He & Zhao, 2020)	*Event driven control strategy	*Resource Consumption	*Estimation error
	*Means of the stochastic Laypunov function	*Communication Overhead	*Data communication rate
	*Stochastic stability theory	*Detection Time	*Sensor battery lifetime.
	*Event driven transmission mechanism		
(Li et al., 2017)	Localization Based	*Localization Error	*Superior in terms of malicious nodes identification and performance improvement.
(Saleem et al., 2016)	Encryption Based	*Resource Consumption	*Efficiently protect WSNs Sybil attacks.
		*Energy Consumption	
		*Computation Overhead	
		*Packet delivery Ratio (PDR)	
		*Computational Cost	

### Hello flood attacks

**Figure 9:**
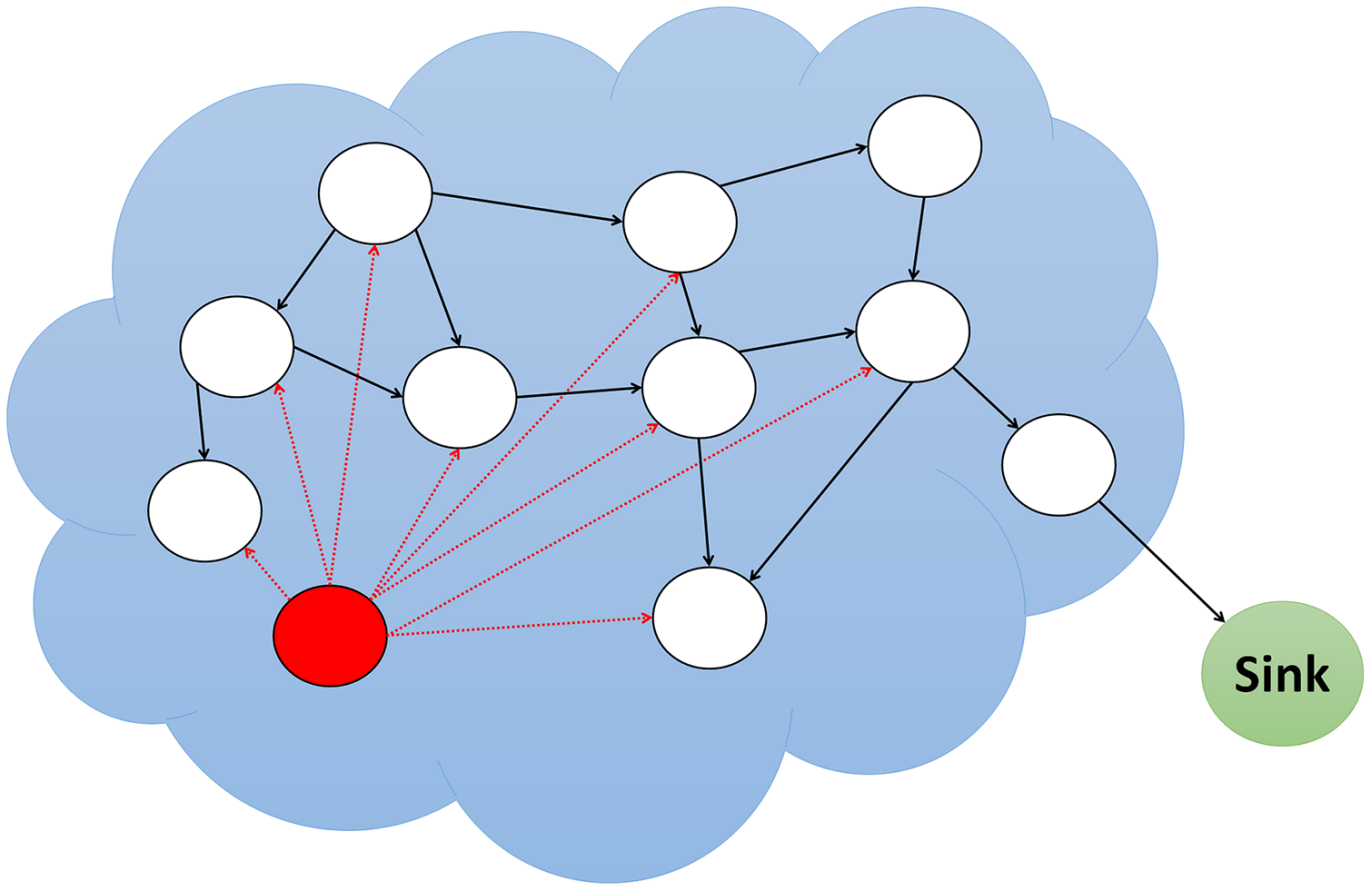
Simulation of hello flood attack.

A Hello Flood attack is one of the more recent attacks on WSNs. In a few protocols, nodes must broadcast Hello packets to let other nodes know they exist. A node that receives a Hello packet assumes that it is within the sender’s node’s radio range. This concept might be untrustworthy, and a laptop-class attacker could convince any network node that the attacker is one of its neighbors. It can only re-distribute overhead packets to the public, as seen in Fig. 9, with the possibility of each network node retrieving them.

The most straightforward defense against a Hello Flood attack is to examine a link on both sides before doing an evocative action on a packet established from a link. Hence, this joint action loses effectiveness when an attacker has a reliable receiver, such as its robust sender. An attacker can effectively create a wormhole in this way. The above method cannot effectively detect and prevent Hello Flood Attacks because the link between these nodes and the attacker is bidirectional. A solution to this problem is that each node authenticates its neighbors with an authentication protocol from a secure base station. If the protocol directs packets in mutual directions of the link, Hello Flood Attacks can be banned when the attacker has a robust transmitter since the protocol checks both directions of the link.

In a multi-step topology, hello flood attacks are typically used to broadcast a packet that every node should receive. The self-organized and decentralized nature of secure, high-sensitivity WSNs poses a significant challenge. It is possible to use global knowledge as a security measure. When the topology is well-formed or altered, or when network scope is constrained. For example, in relatively small WSNs with one hundred nodes or less that have no non-virtual nodes at the development stage, each node can send information to its neighbors and transmit its geographic location to the base station after the initial topology is formed (Sayed & Ashour, 2021). The base station can map the entire network’s topology using the above information. The reason for changing the topology is due to radio interactions or node errors. The nodes renovate a base station with proper information periodically and cause the base station to map the network topology accurately (Khan et al., 2016). Table 8 displays the comparative analysis of currently available hello Flood attack detections in literature.

**Table 8:** HELLO flood attack detections in literature.

Citation	Detection method	Performance evaluation metric	Strengths
(Raghav, Thirugnansambandam & Anguraj, 2020)	Bee algorithm	*Efficiency	*Better data efficiency with security
(Saleem et al., 2016)	Encryption Based	*Resource Consumption	*Efficiently protect WSNs Sybil attacks.
		*Energy Consumption	
		*Computation Overhead	
		*Packet Delivery Ratio (PDR)	
		*Computational Cost	
(Elhoseny et al., 2016)	*Elliptic curve cryptography algorithm	*Network Lifetime	*Much improved network lifetime
	*Encryption method	*Energy Consumption	*Reduced the energy consumption
(Abdus Salam & Halemani, 2016)	Network performance	*End-to-End Delay	Performance in terms of Throughput and delay
		*Throughput	

## Survey methodology

The methodology for Systematic Literature Review (SLR) is illustrated in this section. The researchers conducted an SLR using the instructions provided by the authors with a focus on WSN routing attack detections. Moreover, the research questions and the motivating factors are mentioned in this section. The articles were chosen from the different data sources listed below. A particular search strategy was also classified to find the articles in the domain. The research articles are then carefully analyzed against the inclusion and exclusion criteria listed below before being chosen for review. Table 9 lists the research questions and their rationales to determine the state of the art in routing attack detection in WSN.

**Table 9:** Research questions and motivations.

Questions	Motivations
Q1. What are the limitations of WSN routing attack detections?	The limitations of routing attack detections are addressed in this article.
Q2. What performance evaluation metrics are considered when WSN detects routing attacks?	This article provides the metrics to evaluate the performance of routing attack detection in WSN.
Q3. What are the current research trends and unaddressed issues in WSN?	This article aids researchers in understanding the current state of the art and potential future directions for WSN.

### Data sources

Table 10 lists the articles that were accumulated for review from credible publishers, including Wiley, Tech Science, Springer, ScienceDirect, Sage, MDPI, InderScience, IGI Global, IEEE, Hindawi, Exeley, Elsevier and, Google Scholar.

**Table 10:** Database sources.

Publisher	URL
Wiley	https://wiley.com/
Tech Science	https://www.techscience.com/
Springer	https://springer.com/
ScienceDirect	https://www.sciencedirect.com/
Sage	https://sagepub.com/
MDPI	https://www.mdpi.com/
InderScience	www.inderscience.com
IGI Global	https://www.igi-global.com/
IEEE	https://www.ieee.org/
Hindawi	www.hindawi.com
Exeley	https://www.exeley.com/
Elsevier	https://elsevier.com/
Google Scholar	https://scholar.google.com/

### Search strategy

The focus has been on routing attack detection techniques since 2016. The articles under consideration for this review are from the past 6 years. We define the search words as the first step in figuring out the search string based on the theme and the suggested research questions. The search keywords were “attacks” and “wireless sensor network.” The significant watchwords were associated using the logical operators “AND” and “OR.” After several evaluations, we chose the supplementary search strings that provide an adequate amount of related research. We do this by considering the keywords in Table 11 and framing the search string as follows:

**Table 11:** List of strings and keywords.

String	Batch1 (B1)	Batch2 (B2)
String1 (S1)	Wireless sensor network	Routing Attacks
String2 (S2)	WSN	Attack detection
String3 (S3)	Sensor network	
String4 (S4)	Internet of Things	
String5 (S5)	IoT	

Search Strings: (([B1, S1] OR [B1, S2] OR [B1, S3] OR [B1, S4] OR [B1, S5] AND ([B2,S1] OR [B2, S2])))

### Article selection process

**Figure 10:**
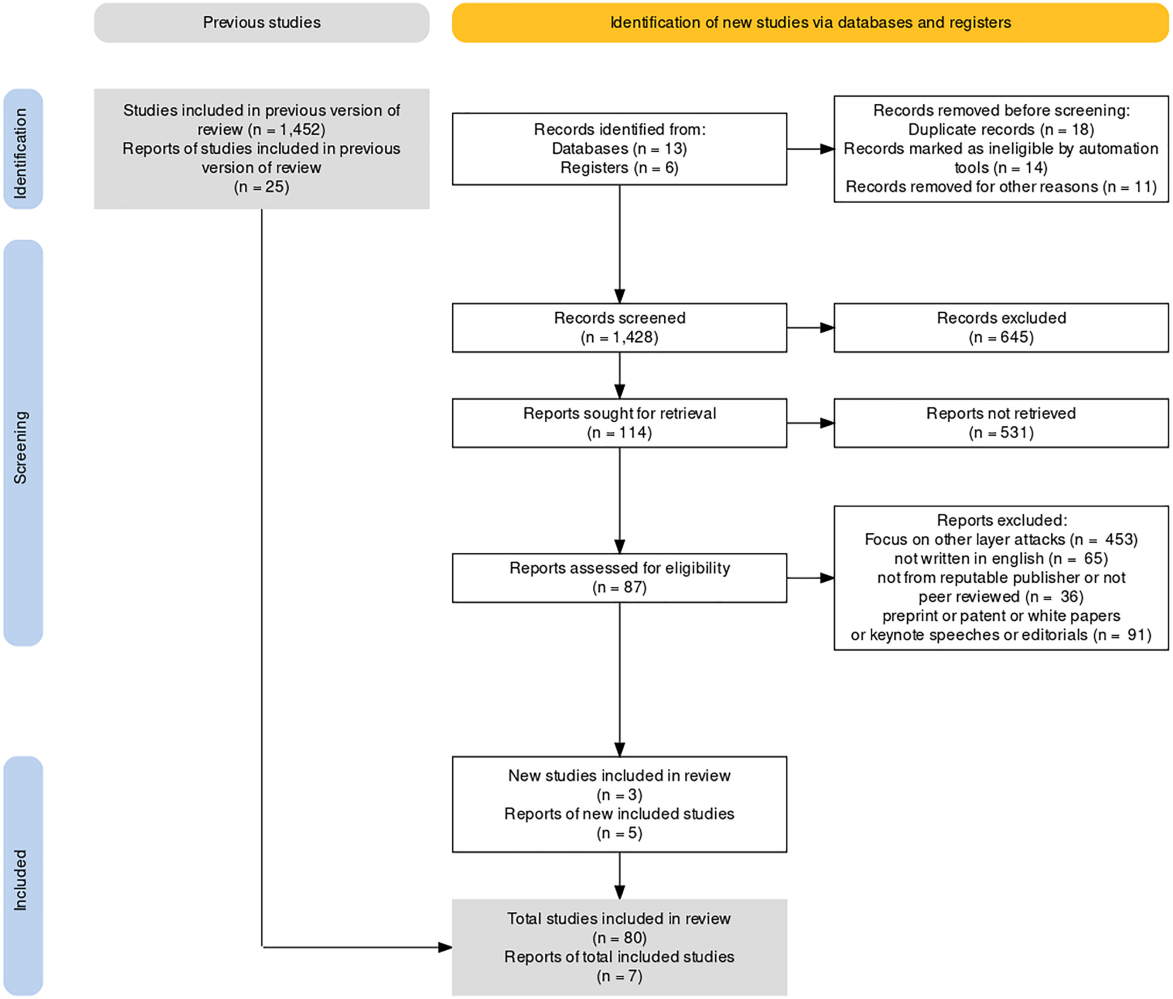
PRISMA flow diagram for article selection.

The research questions are first framed as part of the methodology used in the article selection process. The selection and search processes are aided by structuring the search string. The articles that have been published in English are considered. The scoping review process is conducted under the PRISMA (Prevention and Recovery Information System for Monitoring and Analysis) flow diagram (Peters et al., 2015) to comprehend the most recent advancements and research on detecting routing attacks depicted in Fig. 10. The search process is concluded by categorizing the routing attacks to ensure that this survey is comprehensive. Most of the articles were discarded because their abstracts were not found, or their titles did not meet the screening criteria.

## Results

### Inclusion and exclusion criteria

As shown in Fig. 10, which is a PRISMA flow diagram for article selection, the underlying study generated a total of 1,428 articles from various quality publishers between 2016 and 2022, as mentioned in Table 9. The inclusion and exclusion criteria, listed in Table 12, are implemented to select the significant related research. Therefore, the number of articles was lowered to 783. The number of chosen articles was reduced to 122 based on their titles and abstracts. Following that, 122 articles were examined and thoroughly scrutinized based on the content that matched our classification of routing attack detections in WSN, finally generating 87 articles based on the content. After checking the title, abstract, and comprehensive published research, the essential research articles are selected in accordance with the established criteria to ensure that the findings are relevant to this research article.

**Table 12:** Inclusion and exclusion criteria.

Inclusion criteria	Exclusion criteria
The study focuses on routing attack detections in WSNs.	The articles that focus on other layer attacks.
The articles that are only written in English.	The articles that are not written in English.
The publications from the scholarly publishers and peer-reviewed journals.	The articles that that are not from a reputable publisher or not peer reviewed.
The articles published in WoS and ISI indexed journals.	The articles which are preprint, patents, white articles, keynote speeches, and editorials.

### Year-wise selection

**Figure 11:**
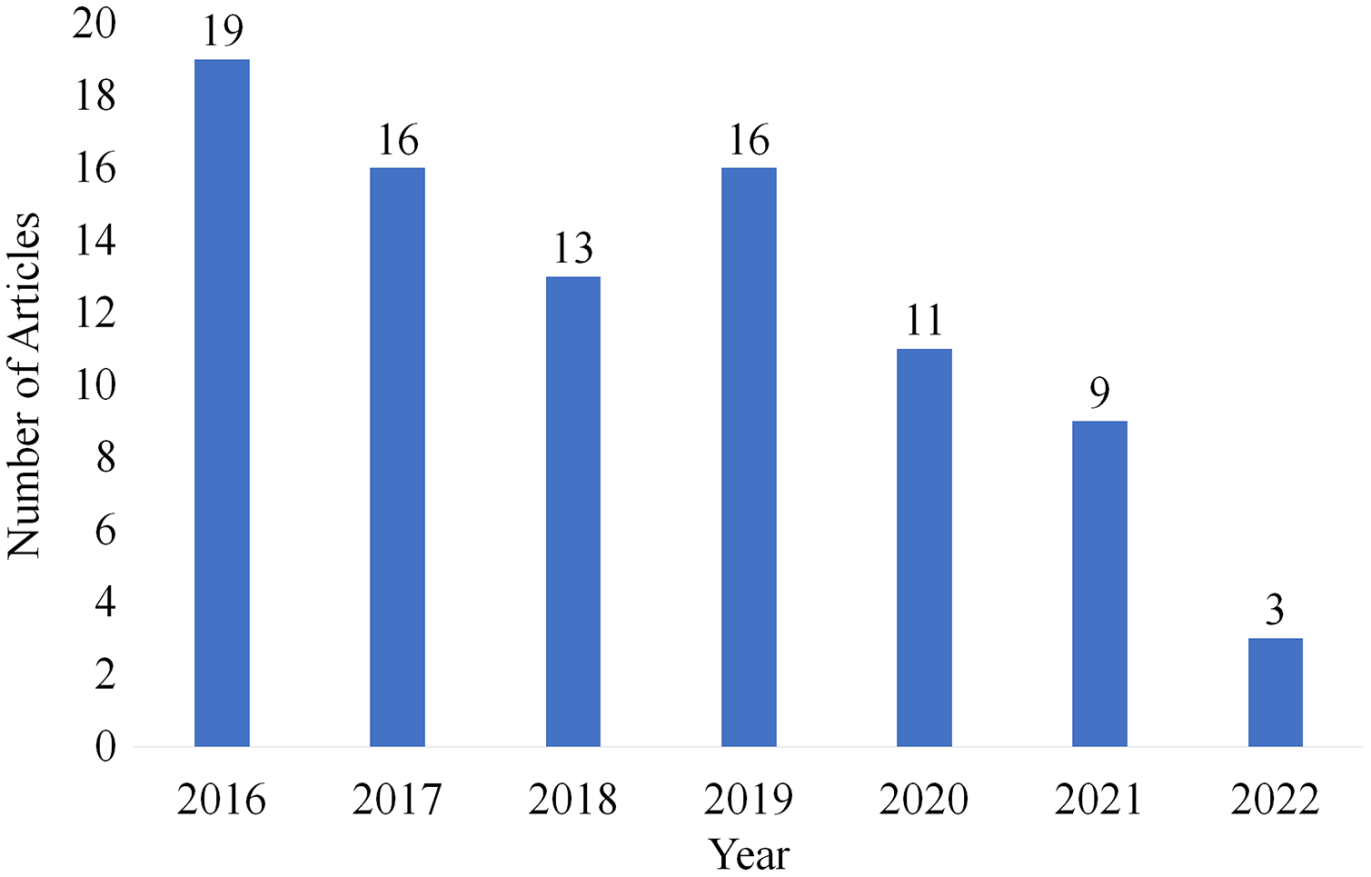
Articles selected for review year-wise.

From the articles which are selected for review, Fig. 11 shows the number of articles published year-wise. To provide a current and relevant literature review, articles from the last 6 years were selected from 2016 to 2022.

### Publisher-wise selection

**Figure 12:**
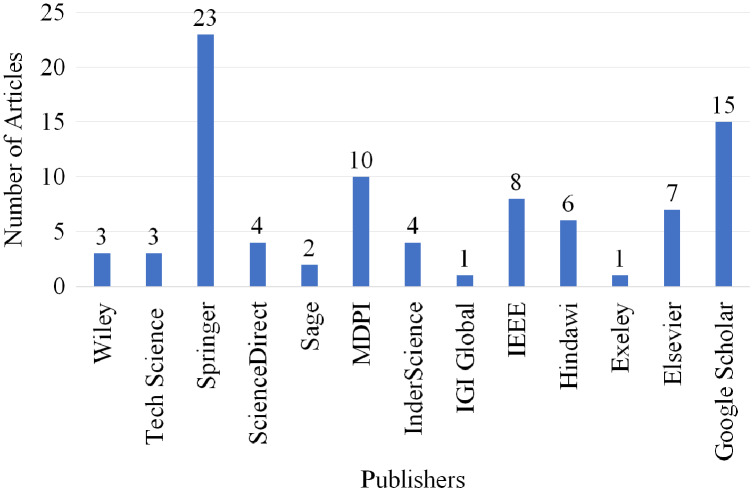
Articles selected for review publisher-wise.

Figure 12 shows the number of articles which were selected and published by well-known scholarly publishers between 2016 and 2022. Overall, of 13 different quality publishers are selected for inclusion of their articles in this SLR article. Three articles are selected from Wiley, three articles are selected from Tech Science, 23 articles are selected from Springer, four articles are selected from ScienceDirect, two articles are selected from Sage, 10 articles are selected from MDPI, four articles are selected from InderScience, one article is selected from IGI Global, eight articles are selected from IEEE, six articles are selected from Hindawi, one article is selected from Exeley and seven articles are selected from Elsevier. Moreover, 15 articles are selected from Google Scholar as it ranks individual articles by considering the publication source, the author, the full text of each document, and the quantity and recency of citations.

### Selection per performance evaluation metric

**Figure 13:**
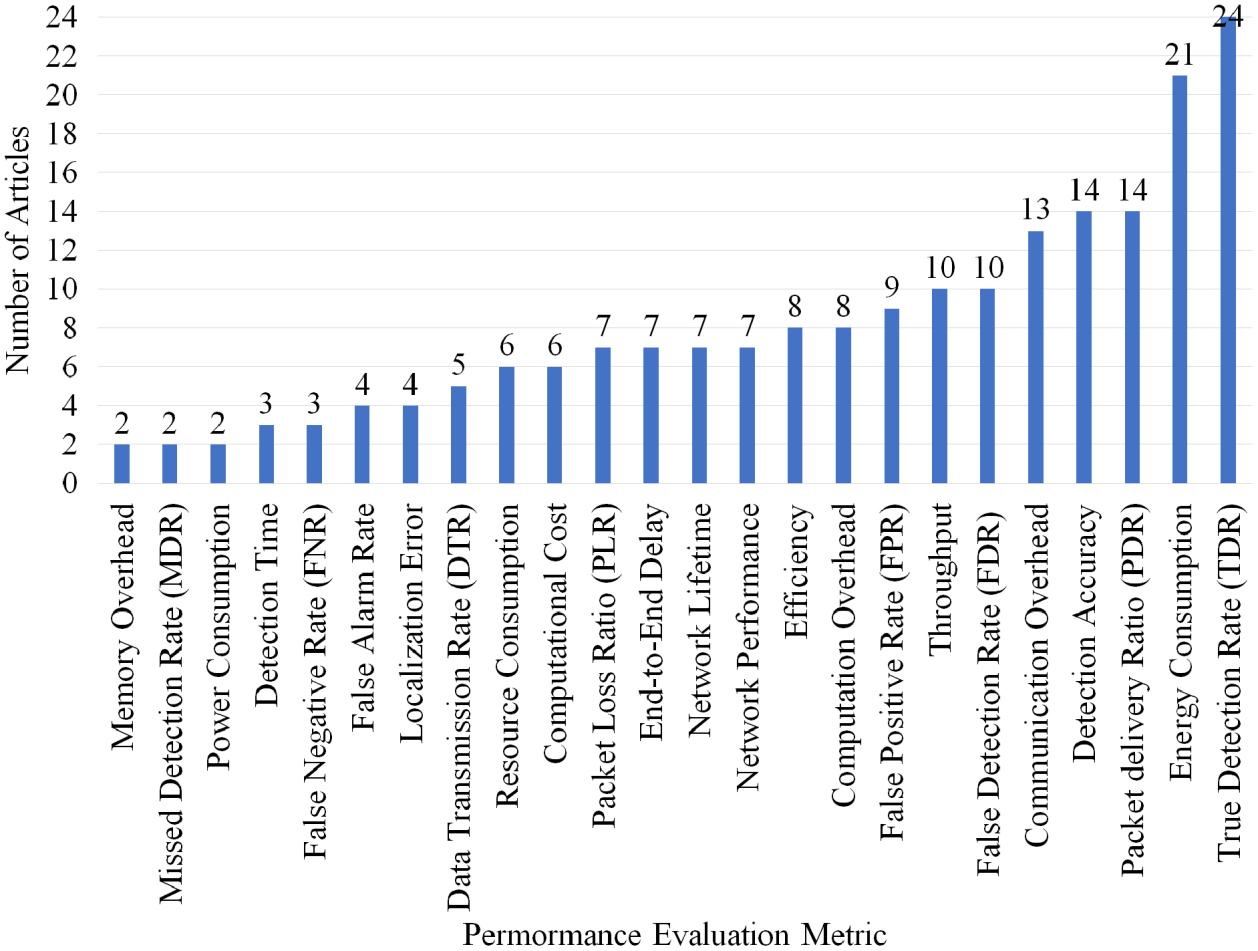
Articles selected for review per performance evaluation metric.

Overall, of 24 different performance evaluations metrics were defined in process of developing this article in which True Detection Rate (TDR), Energy Consumption, Packet delivery Ratio (PDR), Detection Accuracy and communication overhead are the most used metrics in 24, 21, 14, 14 and 13 articles. Figure 13 shows the number of articles used the other metrics.

### Selection per detection method

**Figure 14:**
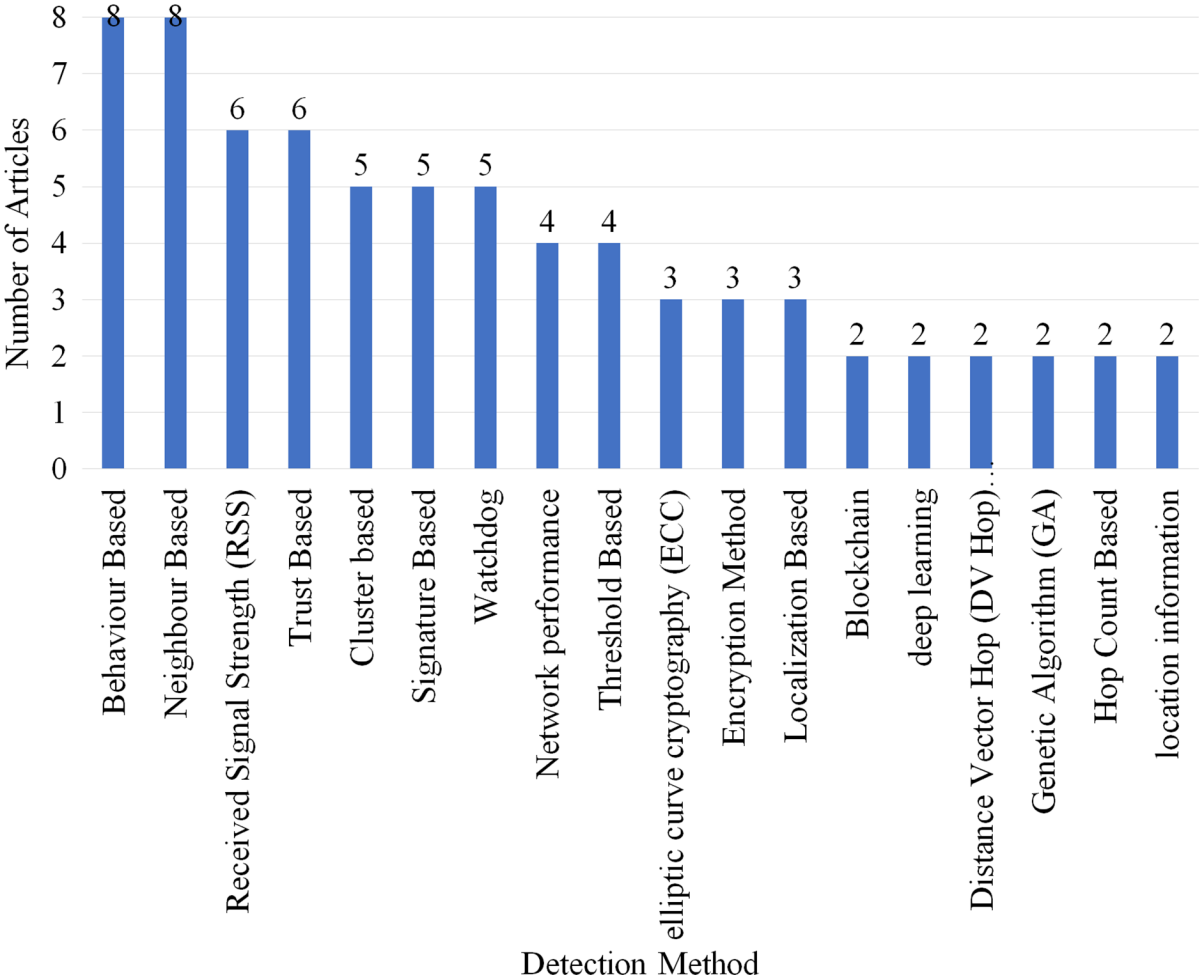
Articles selected for review per detection method.

Figure 14 displays the number of articles which used different detection methods. Total of 79 different detection methods were identified in which only the methods which were used in more than two articles are included in Fig. 14. Eight articles used behaviour-based neighbor-based detection methods which gained the highest rank. RSS and trust-based gained second place as they are used by six articles each.

### Selection per routing attack

**Figure 15:**
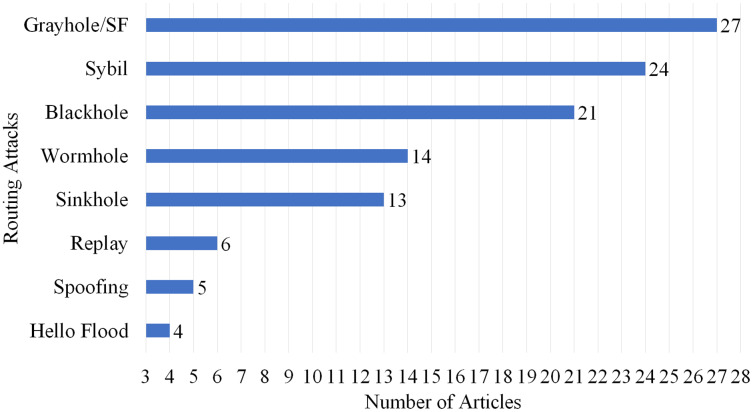
Articles selected for review per routing attack.

This article is the review of 87 different articles from 2016 to 2022 in which some of the articles focused on more than one attack. As per the statistical analysis which is provided in Fig. 15 and the number of articles overviewed the specific attacks, we can sort out the routing attack severity as per the following: wormhole attack (14 articles), Sybil Attack (24 articles), Grayhole/selective forwarding attack (27 articles), blackhole attack (21 articles), sinkhole attack (13 articles), replay attack (six articles), Spoofing attack (five articles) and hello flood attacks (four articles).

### Selection per different metrics for wormhole attack

**Figure 16:**
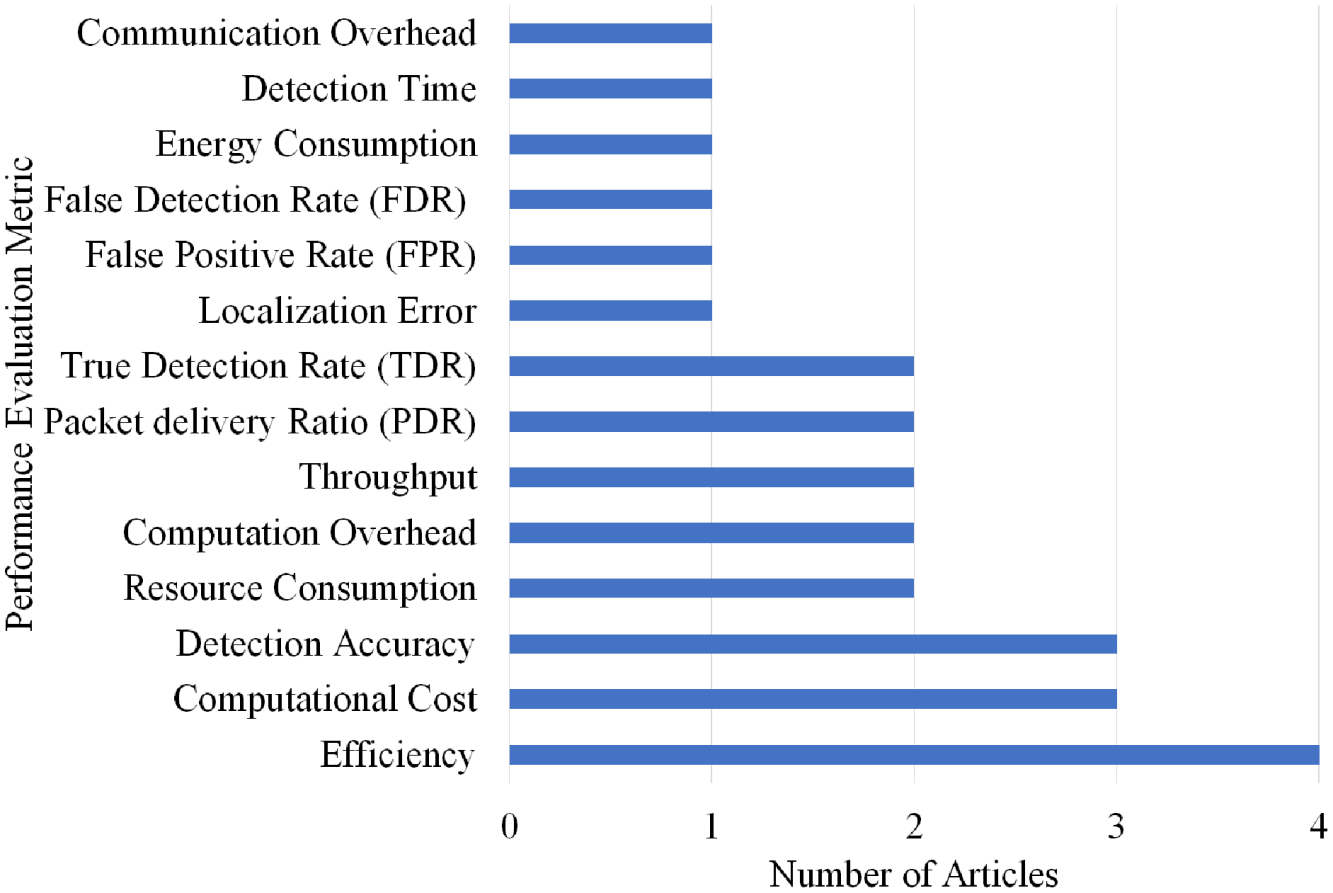
Articles selection based on different metrics used for wormhole attack detection.

Overall, 14 different performance evaluations metrics are used by the articles on wormhole attack detection. As shown in Fig. 16, Efficiency gained the highest rank as it is used by four articles.

### Selection per different metrics for Sybil attack

**Figure 17:**
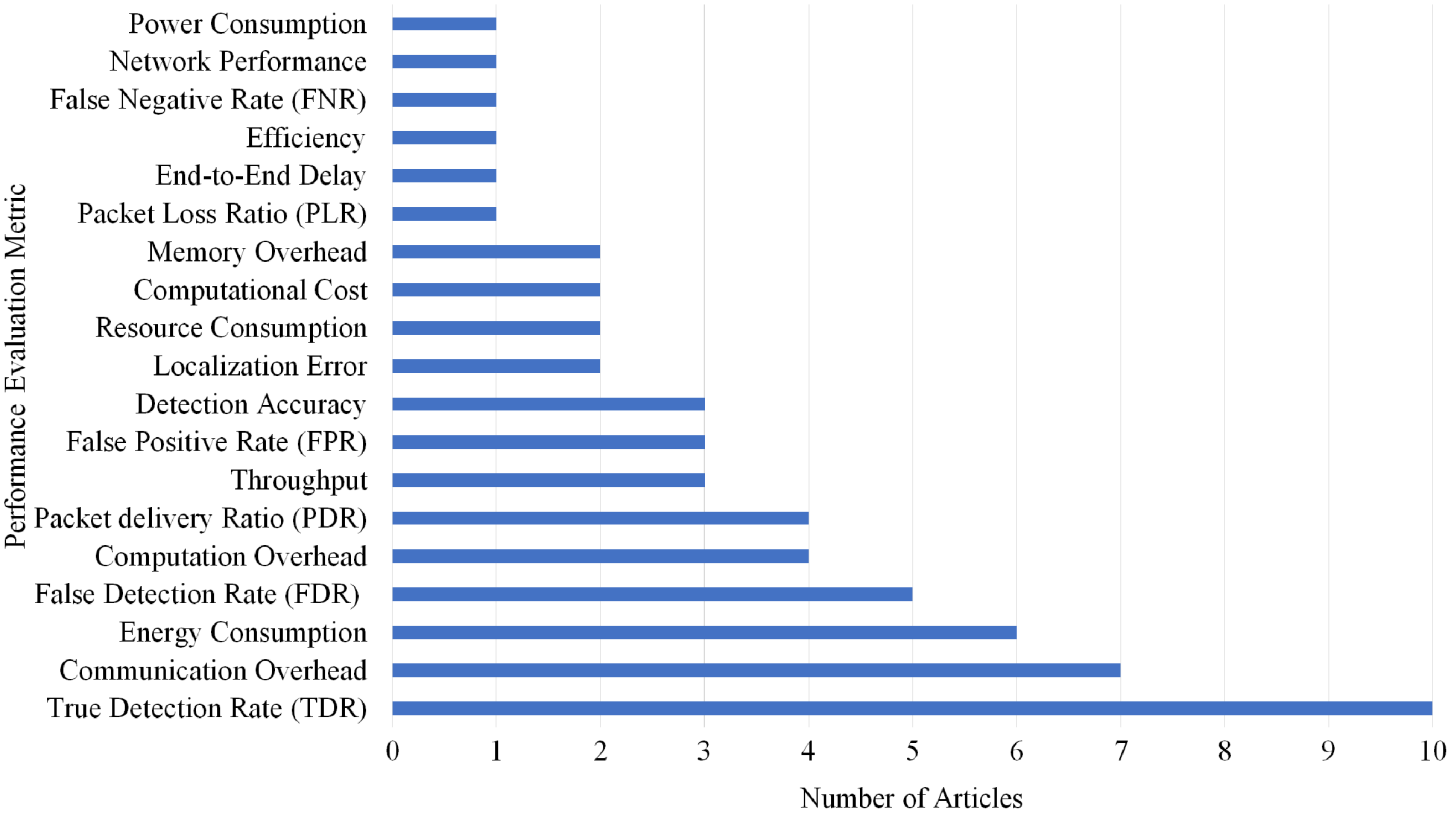
Articles selection based on different metrics used for sybil attack detection.

Overall, 19 different performance evaluations metrics are used by the articles on Sybil attack detection. As shown in Fig. 17, True Detection Rate (TDR) gained the highest rank as it is used by 10 articles.

### Selection per different metrics for Grayhole/SF attack

**Figure 18:**
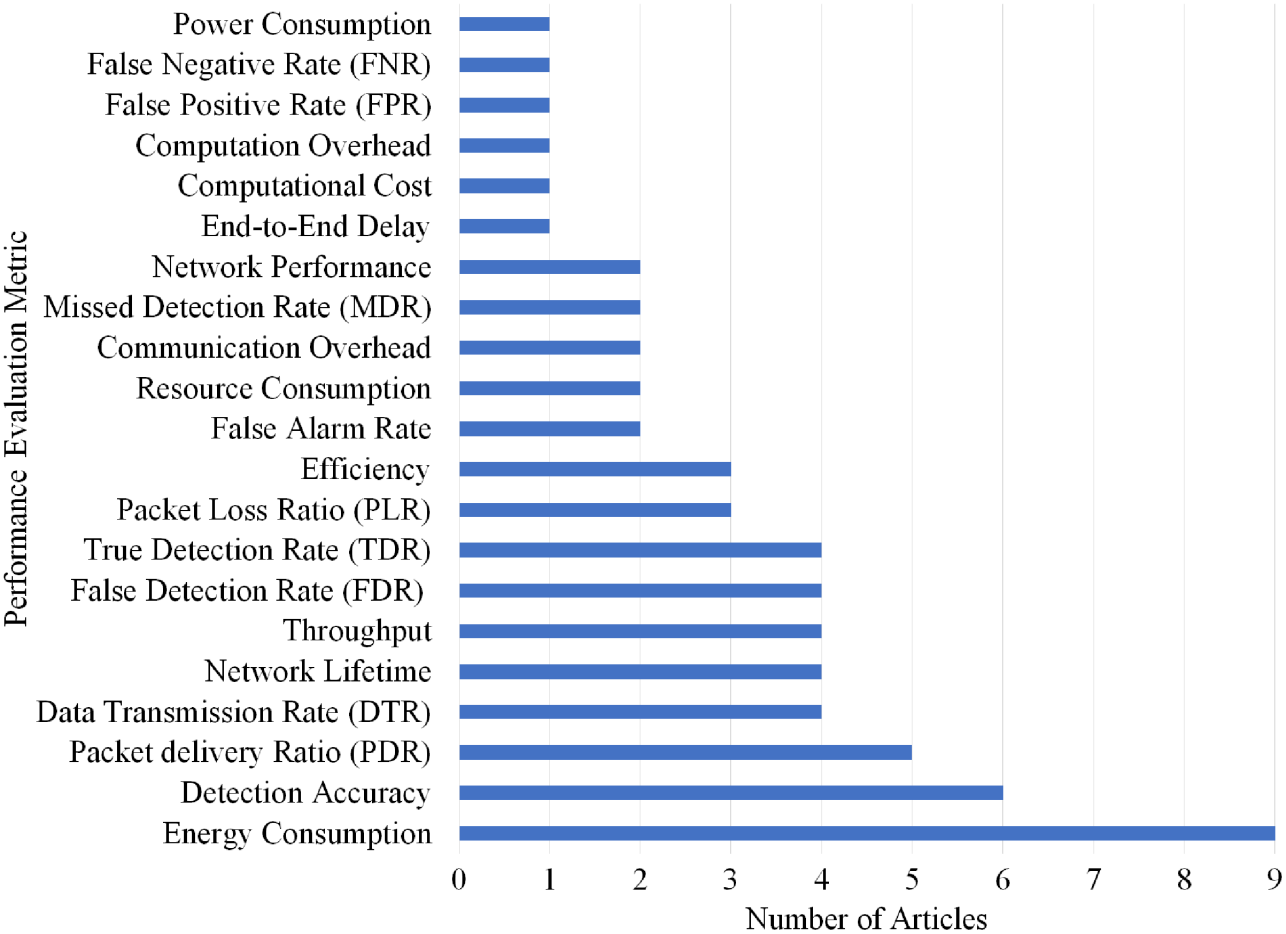
Articles selection based on different metrics used for grayhole/SF attack detection.

Overall, 21 different performance evaluations metrics are used by the articles on Grayhole/SF attack detection. As shown in Fig. 18, Energy Consumption gained the highest rank as it is used by nine articles.

### Selection per different metrics for blackhole attack

**Figure 19:**
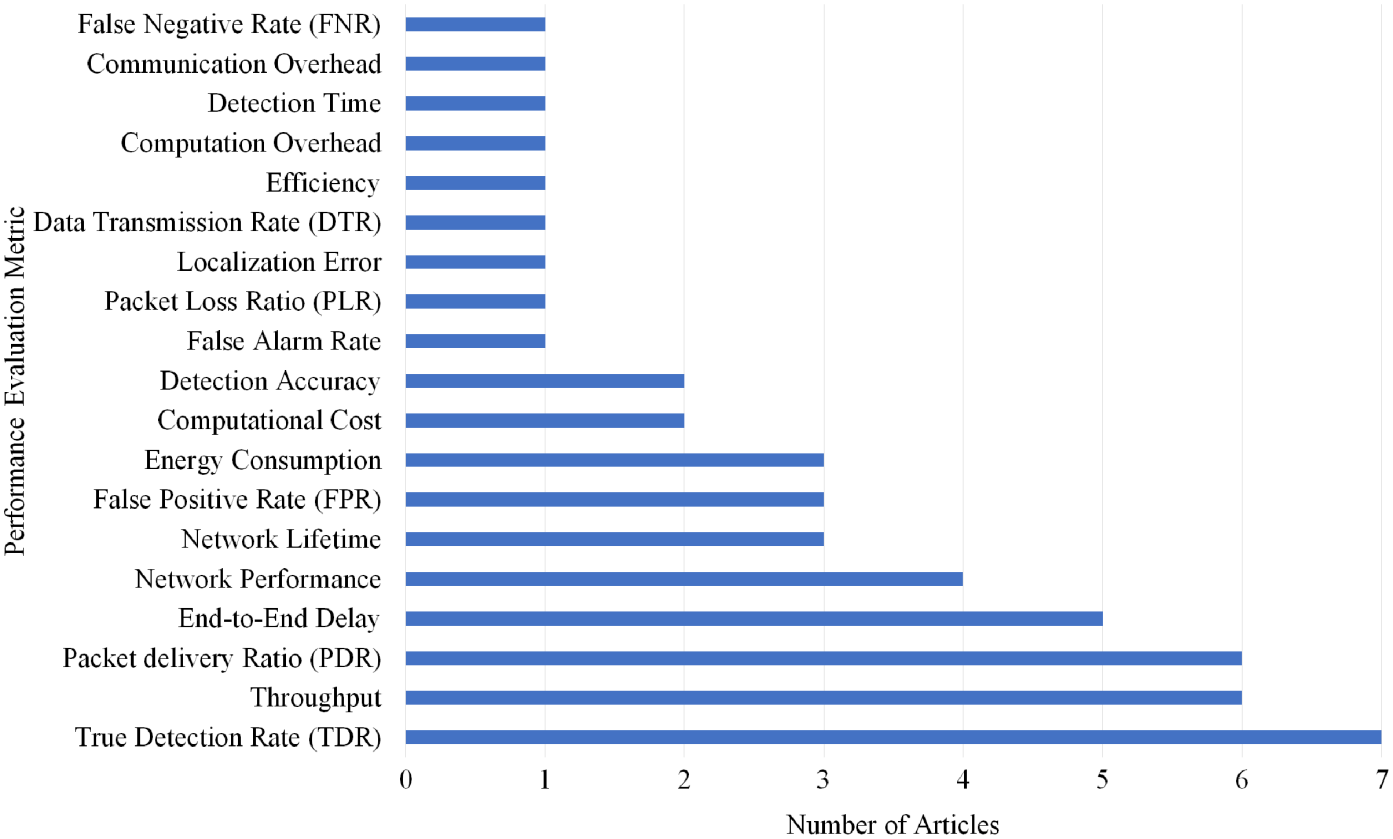
Articles selection based on different metrics used for blackhole attack detection.

Overall, 19 different performance evaluations metrics are used by the articles on Blackhole attack detection. As shown in Fig. 19, True Detection Rate (TDR) gained the highest rank as it is used by seven articles.

### Selection per different metrics for sinkhole attack

**Figure 20:**
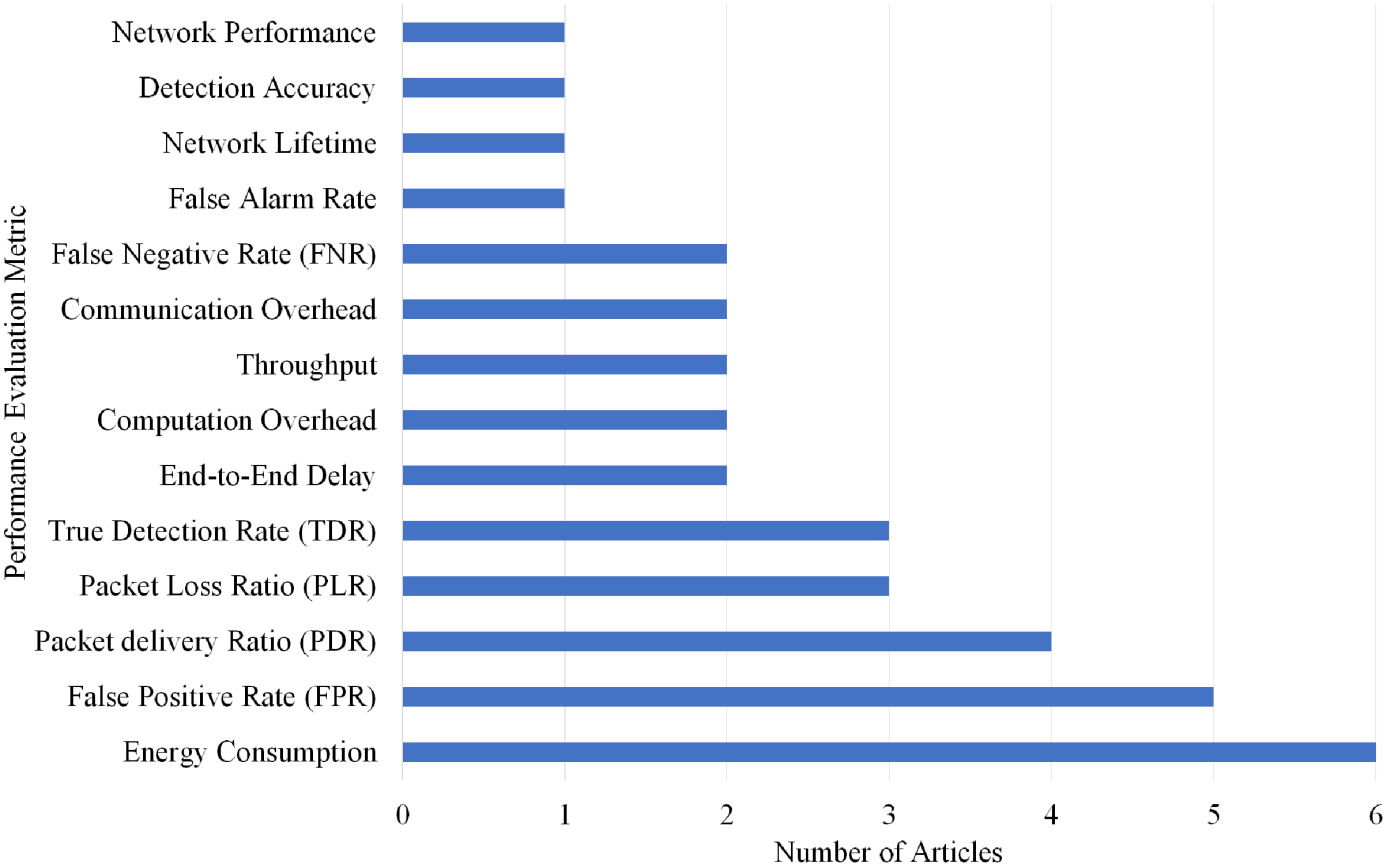
Articles selection based on different metrics used for sinkhole attack detection.

Overall, 14 different performance evaluations metrics are used by the articles on Sinkhole attack detection. As shown in Fig. 20, Energy Consumption gained the highest rank as it is used by six articles.

### Selection per different metrics for replay attack

**Figure 21:**
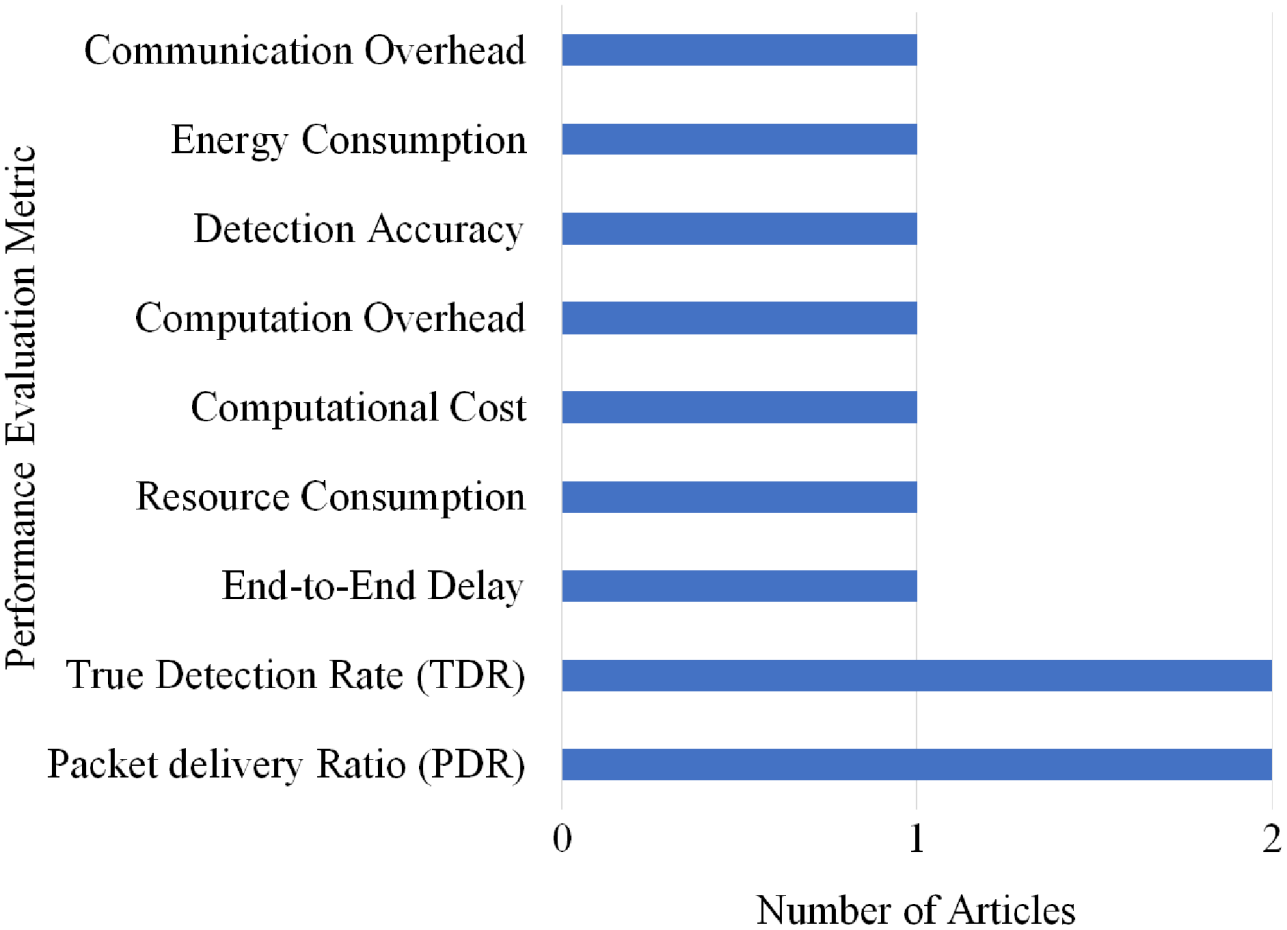
Articles selection based on different metrics used for replay attack detection.

Overall, nine different performance evaluations metrics are used by the articles on Replay attack detection. As shown in Fig. 21, Packet delivery Ratio (PDR) and True Detection Rate (TDR) gained the highest rank as they are used by two articles.

### Selection per different metrics for spoofing attack

**Figure 22:**
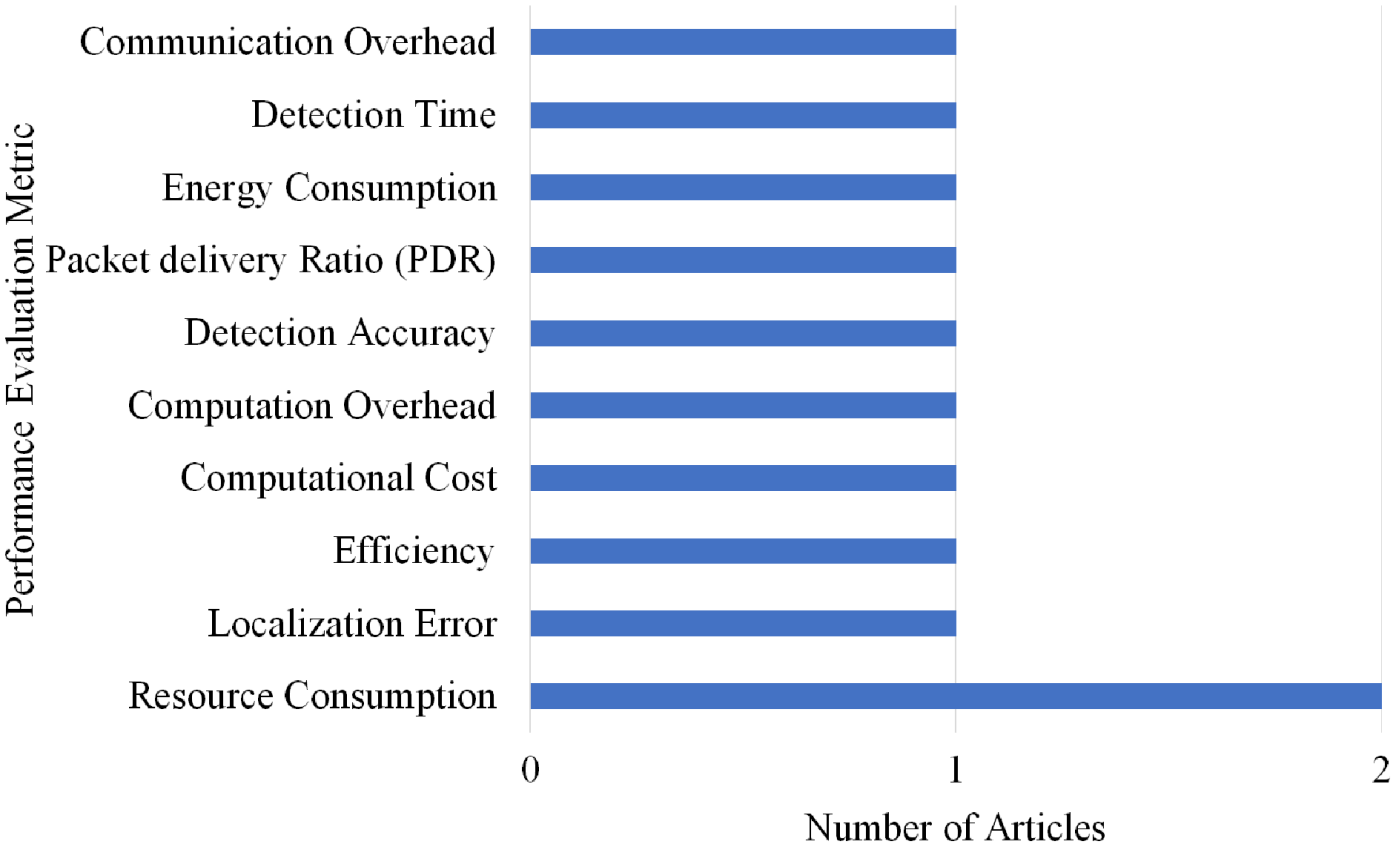
Articles selection based on different metrics used for spoofing attack detection.

Overall, 10 different performance evaluations metrics are used by the articles on Spoofing attack detection. As shown in Fig. 22, Resource Consumption gained the highest rank as it is used by two articles.

### Selection per different metrics for hello flood attack

**Figure 23:**
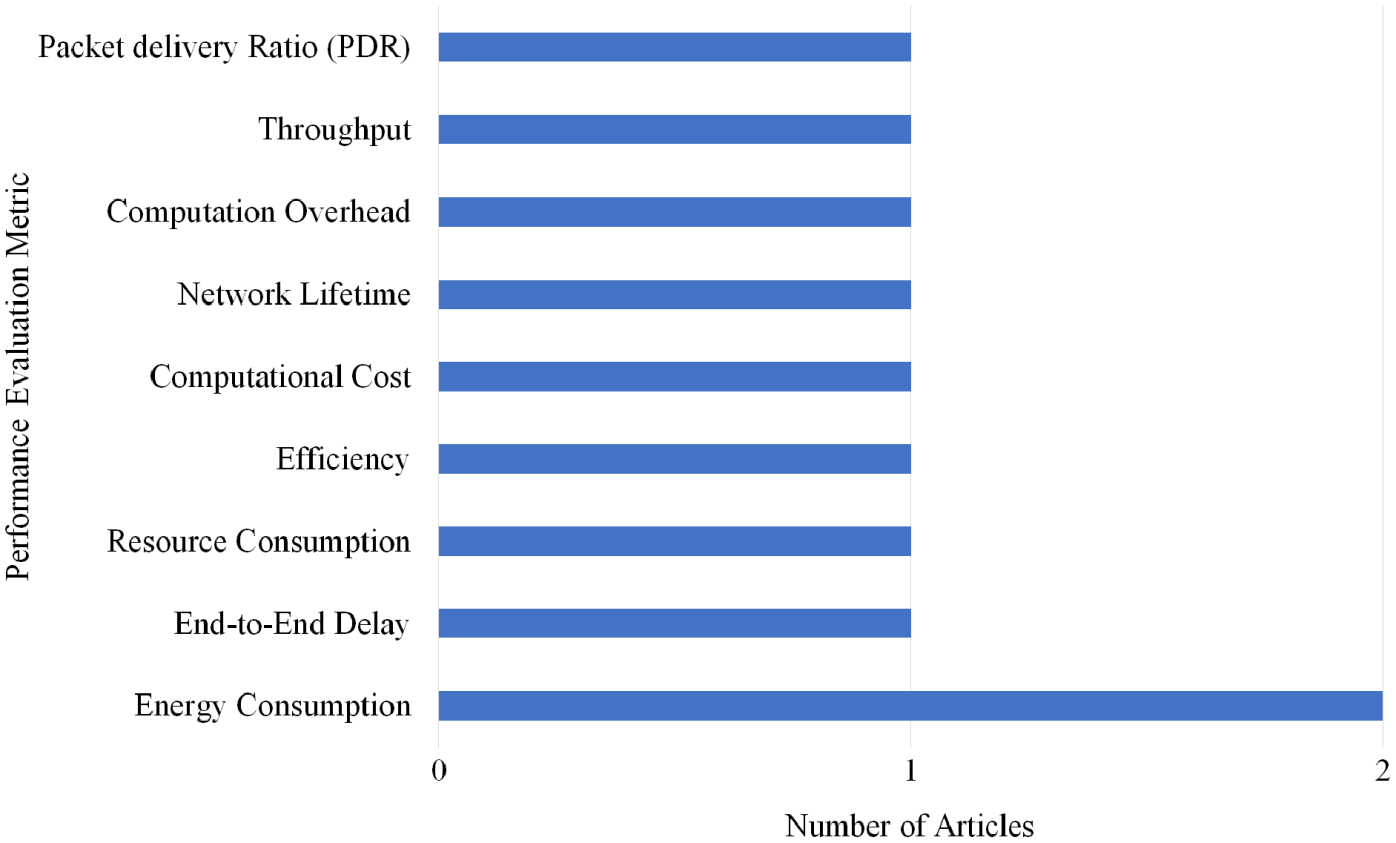
Articles selection based on different metrics used for hello flood attack detection.

Overall, nine different performance evaluations metrics are used by the articles on Hello Flood attack detection. As shown in Fig. 23, Energy Consumption gained the highest rank as it is used by two articles.

## Discussion

The systematic literature review has revealed the following facts and findings against each research question.

Q1. What are the limitations of WSN routing attack detections?

The following are the limitations of WSN routing attack detections which are to be addressed proposing detection techniques for any kind of routing attacks.

### Memory and capacity

Each sensor is a tiny device with a slight volume of memory and storage space for storing the codes (Isidro & Ashour, 2021). With the intention of consuming an efficient detection, it is vital to reduce the code size.

### Energy

It is the most significant constraint for WSNs capabilities. It is assumed that nodes cannot easily insert or recharge after the deployment of WSNs. The impact of the added security code on energy should be taken into consideration when a cryptographic function or protocol is implemented inside the nodes. In other words, when designing a detection tool, it is essential to determine its impact on the node’s lifespan. The extra energy consumed by the nodes is because of the required processing to execute detection, transfer of security-related data, and ultimately safely storing parameters.

### Unknown transmission

In general, communications are wireless because of the packet-based routing in WSNs, and this means the data transfer is uncertain. Packets may be broken due to channel errors or because of network congestion. The result is the loss of the packets. The vital security packets do not get to the correct destination or get lost if protocols are not adequately managed.

### Collision

Even if the channel is reliable, the connection itself may be uncertain. It is due to the nature of the WSNs transmission. If the packets collide in the middle of their way, the transfer operation will fail. In high-density networks, this can be a severe obstacle, which may lead to packets loss.

### Delay

Multi-route routing network congestion (Pulmamidi, Aluvalu & Maheswari, 2021) and processing nodes can lead to increased delays, which may result in a lack of synchronization in WSNs. The synchronization is crucial for WSNs where the detection systems depend on critical event reports or broadcasting cryptographic keys.

### Node seizure attacks

Sensors may be deployed in environments that are accessible to the attacker. So, the probability that a sensor node will be exposed to a physical attack is reasonably more than a server residing in a safer place (Isidro & Ashour, 2021). By taking over the node, it is possible for an attacker to read the valuable information that can include cryptographic keys.

Q2. What performance evaluation metrics are considered when WSN detects routing attacks?

Overall, of twenty-four performance evaluation metrics were identified during the development of this SLR article in which Table 13 displays the articles which use the most 12 common metrics according to different type of attack detection. Figure 13 displays the number of articles for each metrics therefore the most used metrics are: TDR, energy consumption, PDR, detection accuracy, communication overhead, FDR, throughput, FPR, computation overhead, efficiency, network lifetime and end-to-end delay.

**Table 13:** Performance evaluation metric per attack.

Article	Attack	True detection rate (TDR)	Energy consumption	Packet delivery ratio (PDR)	Detection accuracy	Communication overhead	False detection rate (FDR)	Throughput	False positive rate (FPR)	Computation overhead	Efficiency	Network lifetime	End-to-End Delay
(Jamshidi et al., 2019a)	Sybil	*				*	*			*			
(Wazid et al., 2016)	Sinkhole	*				*			*	*			
(Razaque & Rizvi, 2017)	*Sybil *Sinkhole		*		*	*							
(Jamshidi et al., 2019c)	Sybil	*				*	*						
(Zhang et al., 2019)	Sinkhole	*	*						*				
(Raja & Beno, 2017)	Sybil		*	*				*					
(Ariffin, Mokhtar & Abd Rahman, 2018)	Blackhole		*	*								*	
(Wazid & Das, 2016)	Blackhole	*							*			*	
(Yadav & Mishra, 2020)	Blackhole			*				*					*
(Reji et al., 2017)	Sinkhole		*							*			
(Patel, Aggarwal & Chaubey, 2018)	Wormhole				*					*			
(Yuan et al., 2018)	Sybil	*				*							
(Derhab et al., 2020)	Grayhole/SF		*		*								
(Farooqi & Khan, 2017)	*Grayhole/SF *Sinkhole *Blackhole		*					*					
(Elhoseny et al., 2016)	*Grayhole/SF *Hello Flood		*									*	
(Nithiyanandam & Parthiban, 2020)	Sinkhole		*									*	
(Mathur, Newe & Rao, 2016)	*Grayhole/SF *Blackhole	*			*								
(Jamshidi et al., 2017)	Sybil	*					*						
(Jamshidi et al., 2019b)	Sybil	*					*						
(Wazid & Das, 2017)	Blackhole	*							*				
(Ren et al., 2016)	Grayhole/SF			*	*								
(Vaniprabha & Poongodi, 2019)	*Wormhole *Sinkhole *Sybil			*					*				
(Kumar et al., 2016)	*Wormhole *Blackhole *Sybil			*				*					
(Bilgin & Baktir, 2019)	*Blackhole *Replay			*									*
(Abdus Salam & Halemani, 2016)	Hello Flood							*					*
(Zhu et al., 2018)	*Grayhole/SF *Blackhole					*							
(Wen & Wang, 2018)	Replay					*							
(Yuan & Wang, 2020)	Sinkhole		*										
(Singh & Saini, 2021b)	Sybil		*										
(Shu et al., 2017)	Wormhole		*										
(Karakoç & Çeken, 2021)	*Blackhole *Replay	*											
(Alqahtani et al., 2019)	*Grayhole/SF *Blackhole	*											
(Pawar & Jagadeesan, 2021)	*Wormhole *Blackhole	*											
(Gara, Ben Saad & Ben Ayed, 2017)	Grayhole/SF	*											
(Garcia-Otero & Poblacion-Hernandez, 2016)	Replay	*											
(Wang & Feng, 2020)	Sybil	*											
(Singh, Singh & Singh, 2016a)	Sybil	*											
(Wang, Wen & Zhao, 2018)	*Sybil *Replay				*								
(Huan, Kim & Zhang, 2021)	Spoofing				*								
(Li & Cheffena, 2019)	Sybil				*								
(Lal & Prathap, 2021)	Grayhole/SF							*					
(Raghav, Thirugnansambandam & Anguraj, 2020)	*Sybil *Hello Flood *Spoofing										*		
(Garcia-Font, Garrigues & Rifa-Pous, 2017)	Grayhole/SF										*		
(Yi et al., 2018)	Grayhole/SF										*		
(Lai, 2016)	Wormhole										*		
(Singh et al., 2018)	Wormhole										*		
(Mukherjee et al., 2016)	Wormhole										*		

Q3. What are the current research trends and unaddressed issues in WSN?

In recent years, research on WSNs security has become more prominent. In the design of WSNs, there are several factors and open research issues that need to be considered.

### Hardware

Each node must be small enough, lightweight, and low volume while having all the necessary components. For example, in some applications, the node should be as small as a matchbox; sometimes, the node’s size is limited to one cubic centimeter (Alansari et al., 2021). It should be light enough to hang in the air with the wind in terms of weight. At the same time, each node must have minimal power consumption, low cost, and be compatible with environmental conditions. These are all limitations that challenge the design and construction of sensor nodes.

### Connectivity

A sensor network has graph connectivity as its inbuilt connectivity. Each node has connections to several other nodes in its vicinity because of the nodes’ wireless connections and public broadcasting. Efficient algorithms for collecting data and applications for tracking network objects, such as spanning trees, are considered (Siddiqui & Ashour, 2021). Hence, the traffic is such that the data travels from some node to another; connectivity management should be done carefully. An essential step in the management of network connectivity is the initial setup. Nodes that have not had any initial communication before should be able to communicate with one another once they are hired and started to work. Connectivity management algorithms should be able to subscribe to new nodes in the initial setup and removes the nodes which do not work for any reason. The connectivity dynamics of the sensor network properties are an issue that challenges security. Providing dynamic connectivity management methods that can cover security issues is one of the great ideas for future studies.

### Reliability

Each node can be broken or destroyed entirely by environmental events, such as an accident or explosion, or can fail when the energy source is exhausted (Alansari, Siddique & Ashour, 2022). The purpose of tolerance or reliability is that node failure should not affect the overall network performance and build a reliable network using unreliable components.

### Scalability

The network should be scalable concerning the number of nodes and the allocation of nodes. In other words, the sensor network should be able to work with hundreds, thousands, and even millions of nodes and support the density of different nodes’ distribution. In several applications, nodes are randomly distributed, and environmental factors displace no possibility of distribution with a specific and uniform density of nodes. As a result, the density should be flexible, ranging from a few to one hundred nodes. The various approaches also have an impact on the scalability problem. For instance, they will not work in a specific density or with a certain number of nodes. Specific techniques, on the other hand, are scalable.

### Overall cost

As the number of nodes is high, each node’s cost reduction is critical. The number of nodes sometimes reaches millions which, in this case, the cost reduction of the node, even in small quantities, has a significant effect on the total price of the network.

### Environmental conditions

A wide range of WSNs applications is related to environments in which humans cannot be present. Like chemical, microbial, or nuclear-contaminated environments or underwater studies, space, or military environments due to the presence of the enemy. In the forest and Inhabitants of animals, the presence of human beings will escape them. In each case, the environmental conditions should be considered in the design of the nodes. For example, in the sea or wet environments, the sensor node must be placed in a chamber that does not transmit moisture.

### Communication media

In WSNs, nodes communicate wirelessly through radio media, Infrared Radiation (IR), or other optical media. The infrared connection is cheaper and easier to build, but it only works in a direct line.

### Power consumption of nodes

The WSNs nodes must have low power consumption. Sometimes the power supply of a battery is a 1.2 V with a flow of 0.5 amps per hour, which should provide the necessary power for a long time, for example, nine months. In many applications, the battery is not replaceable. Therefore, battery life establishes the life of the node. Moreover, a node acts as a pathfinder, receiving information (by the sensor) or running a command (by the actuator). Faulty operation of a node removes it from the connections and would cause network reorganization and rerouting of the packets (Alansari, Prasanth & Belgaum, 2018). Designing the node’s hardware, using low power consumption components, and providing the possibility of a sleep mode for the entire node or each section is essential.

### Increasing the network lifetime

WSNs typically have a short lifetime due to the nodes’ insufficient power supply. Additionally, a network node’s location can occasionally exacerbate the problem. For instance, a node only one hop away from a sink quickly runs out of energy from an excessive workload. On the other hand, if it fails, the sink will be disconnected from the entire network, and WSNs will stop working. Some solutions involve the network structure; for example, an automated structure is a great way to address the problem. The automated structure makes most of its decisions locally (Maheswari, Raju & Reddy, 2019). As a result, the node’s and network’s lifetime increase even though the transmission traffic through it decreases. Any WSN with an uneven node distribution will experience the issue of early energy depletion on nodes with low-density regions. To ensure that crucial nodes are used as less as possible, it would be appropriate in these circumstances to implement power management within the nodes and provide some power awareness solutions. Since distributed nodes in the sensor/actuator field share resources, effective task management, and power management will lengthen the network lifetime. Providing appropriate structural patterns, management methods, and intelligent power algorithms to grow the network lifetime worth further investigation.

### Real-time communication and co-ordination

In some applications, the network response speed is crucial such as the system for detecting and preventing the spread of fire or theft prevention system. The packets must be instantaneously updated in the immediate display of pressure on the monitor. A way to realize the system’s real-time connectivity is to set a deadline for packets. In the media access control layer, packets with the shortest deadlines will be sent sooner. The duration of the cut-off depends on its application. Respectively, another critical issue is event report delivery to the sink in order of occurrence. Otherwise, the network may not respond appropriately. One more issue is the coordination of the network with the related reports given to the sink of a specific area in case of an event. For example, assume in a military application that some sensors to detect the occurrence of enemy units and some tools to destroy them are considered. Several sensors inform the sink of the presence of an enemy. The network must start the operation in the entire area immediately; otherwise, with the response of the first sink, the enemy soldiers are dispersed, and the operation is defeated. However, the issue of instant communication and coordination in sensor networks, especially in large-scale and uncertain conditions, is still a topic of research.

### Unpredictable factors

WSNs are a function of many uncertainties. Unpredictable natural factors such as floods, earthquakes, problems caused by wireless communication and radio disturbance, node failure, sensor failure, dynamics structure, network routing, the addition of new nodes and the removal of old nodes, automated nodes replacement, or by natural factors. The issue is how to develop, from a network layer perspective, an outlook in such a situation that is a solid, large-scale entity with a reliable operational capability which will be addressed in this SLR.

### Limitations of this SLR

- This review only took a limited set of databases and journals into account.
- Additionally, only a few keywords and string combinations have been used to search the literature.
- This review has not considered any articles that were published prior to 2016.
- This review primarily focuses on routing attack detections rather than application, transport, datalink, or physical layer attacks on WSNs.

## Conclusions

This article provides a systematic literature review of routing attack detection methods and metrics used by 87 articles from 2016 to 2022 using a PRISMA flow diagram. The selected articles are based on eight routing attacks: wormhole, Sybil, Grayhole/selective forwarding, blackhole, sinkhole, replay, spoofing, and hello flood attacks. Although the impact of routing attacks over WSNs manifested over recent years, inconsiderable attention was given to implementing decent routing attack detection. The outcomes of this study designated that different routing attack detection techniques and algorithms can be successfully employed on WSNs. Consequently, the study has endowed new tendencies and potentials for future researchers. This study allows wireless sensor network administrators, service providers, and end-users to undertake additional research in the future to improve the security of WSNs.

Having a clear goal and foresight in any field can significantly contribute to advancing technology in that field. In the future, we aim to introduce some techniques that can be used by researchers interested in WSNs and the security dimension of these networks. Introducing new and combined methods can get better results and enhance the security of WSNs, such as:

- The use of node clustering or distributing techniques in the network and the use of hedge mechanisms to provide new methods in the critical management area.
- The use of elliptical bending encryption math for efficiently swapping keys between network nodes.

## Supplemental Information

10.7717/peerj-cs.1135/supp-1 Supplemental Information 1 87 articles breakdown which were used for SLR.

## Additional Information and Declarations

### Data Availability

The following information was supplied regarding data availability: The raw measurements are available in the Supplemental File.
